# Oxygen Modulates the Effectiveness of Granuloma Mediated Host Response to *Mycobacterium tuberculosis*: A Multiscale Computational Biology Approach

**DOI:** 10.3389/fcimb.2016.00006

**Published:** 2016-02-15

**Authors:** Cheryl L. Sershen, Steven J. Plimpton, Elebeoba E. May

**Affiliations:** ^1^Department of Biomedical Engineering, University of HoustonHouston, TX, USA; ^2^Center for Computing Research, Sandia National LaboratoriesAlbuquerque, NM, USA

**Keywords:** *Mycobacterium tuberculosis*, agent based model, systems biology, granuloma, multiscale modeling, host-pathogen interactions, dormancy, lung diseases

## Abstract

*Mycobacterium tuberculosis* associated granuloma formation can be viewed as a structural immune response that can contain and halt the spread of the pathogen. In several mammalian hosts, including non-human primates, *Mtb* granulomas are often hypoxic, although this has not been observed in wild type murine infection models. While a presumed consequence, the structural contribution of the granuloma to oxygen limitation and the concomitant impact on *Mtb* metabolic viability and persistence remains to be fully explored. We develop a multiscale computational model to test to what extent *in vivo Mtb* granulomas become hypoxic, and investigate the effects of hypoxia on host immune response efficacy and mycobacterial persistence. Our study integrates a physiological model of oxygen dynamics in the extracellular space of alveolar tissue, an agent-based model of cellular immune response, and a systems biology-based model of *Mtb* metabolic dynamics. Our theoretical studies suggest that the dynamics of granuloma organization mediates oxygen availability and illustrates the immunological contribution of this structural host response to infection outcome. Furthermore, our integrated model demonstrates the link between structural immune response and mechanistic drivers influencing *Mtb*s adaptation to its changing microenvironment and the qualitative infection outcome scenarios of clearance, containment, dissemination, and a newly observed theoretical outcome of transient containment. We observed hypoxic regions in the containment granuloma similar in size to granulomas found in mammalian *in vivo* models of *Mtb* infection. In the case of the containment outcome, our model uniquely demonstrates that immune response mediated hypoxic conditions help foster the shift down of bacteria through two stages of adaptation similar to the*in vitro* non-replicating persistence (NRP) observed in the Wayne model of *Mtb* dormancy. The adaptation in part contributes to the ability of *Mtb* to remain dormant for years after initial infection.

## Introduction

Tuberculosis (TB) disease, caused by the bacilli *Mycobacterium tuberculosis* (*Mtb*), remains a major global health concern, with an estimated 8.6 million infected globally and 1.2 million *Mtb* related deaths in 2012 (World Health Organization, 2013). After inhalation of *Mtb* in the form of microdroplet nuclei, the bacteria are phagocytized by lung alveolar macrophages. An initial innate immune response ensues followed by presentation of *Mtb* antigens by professional antigen presenting cells (e.g., macrophage and dendritic cells) to lymphocytes, leading to cell-mediated immune response. Immune response to *Mtb* infection is characterized by sequential recruitment of leukocytes such as T, B, and NK cells as well as uninfected macrophages to the site of infection (Co, 2004). In the event that immune cells are unable to eliminate the infection (clearance scenario), these cells attempt to contain the spread of infection by aggregating in multiple layers around the infected host cell leading to the formation of granulomatous structures (containment scenario). In the event, that the host fails to clear or contain the pathogen, *Mtb* can spread, infecting other cells, tissues, and organs (dissemination scenario).

A granuloma can be viewed as an equilibrium state where the host contains the infection while the pathogen persists by transitioning into a dormant or latent state, resulting in latent TB infection (LTBI). The WHO estimates one-third of all individuals have LTBI, with the risk of reactivation to active disease ranging from 5 to 20% depending on the health of the individual (World Health Organization, 2013). Within human pulmonary macrophages and granuloma structures, *Mtb* is believed to be in a microenvironment that has diminished oxygen availability and increased nitric oxide (NO) concentrations (Gomez and McKinney, 2004; Shiloh et al., 2008). The physical structure of the granuloma with a central focus of *Mtb* infection surrounded by multiple layers of epithelioid cells and a mantle of lymphocytes is likely a key contributing factor to the depletion of oxygen in the *in vivo* microenvironment of *Mtb* (Via et al., 2008). How oxygen depletion within the granuloma microenvironment influences *Mtb* proliferation and persistence is important to understanding and ultimately treating LTBI.

The role of host response mediated oxygen depletion on *Mtb* survival has been the focus of several empirical studies, most notably the Wayne model of non-replicating persistence (NRP) which provided an *in vitro* platform for analyzing *Mtb*s metabolic response to oxygen depletion (Wayne and Hayes, 1996; Voskuil et al., 2004; Shiloh et al., 2008; Deb et al., 2009). To effectively treat *Mtb* and LTBI, increased understanding of the multiscale mechanistic impact of host biochemical and physiological immune response on *Mtb* metabolic viability is necessary to identify possible molecular targets that impact infection outcome. In this work, we focus on understanding the contribution of the granuloma in dynamic modulation of the microenvironment of *Mtb*, and *Mtb*s response as evidenced by the pathogens consequential elimination, containment, or dissemination.

While there are well-established computational models of infection, most notably Segovia-Juarez et al. (2004) and Ray et al. (2009), the majority of existing models do not explicitly consider or investigate the role of oxygen on infection outcome and disease. Integrating *in vitro* and *in vivo* empirical data, several agent based models (ABM) of tuberculosis infection have been developed and used to capture the spatio-temporal dynamics of granuloma formation in humans and the impact of TNF-α (tumor necrosis factor) on *Mtb* within a single granuloma (Segovia-Juarez et al., 2004; Warrender et al., 2006; Ray et al., 2009; Fallahi-Schani et al., 2011; Marino et al., 2011). In these models of TB infection, cellular entities (macrophages at various stages of infection, inflammatory T cells, cytotoxic T-lymphocytes, T regulatory cells) are represented as discrete elements or agents. Chemokines, cytokines such as TNF-α, and extracellular *Mtb* are modeled using continuous valued fields. Recent extensions to the TB ABM models include the expansion of the macrophage rule based model into a systems biology based model that includes the signal transduction mediated response of the host to cytokines in the extracellular compartment (Cilfone et al., 2013). An advancement needed in the ABM modeling approach is incorporation of variables describing physiological changes in the lung parenchyma and explicit consideration of *Mtb* biochemical dynamics to capture the metabolic response of the pathogen to the host modulated microenvironment.

Recently Datta et al. (2015) developed an empirical-based model of an idealized granuloma that captures oxygen transport and consumption using Michaelis–Menten based kinetic approximations. Their model was used to predict the size and shape of granulomas, and outcomes were comparable to *in vivo* rabbit models of disease. However, to our knowledge there is not yet an *in silico* study that fully links host physiological response and oxygen availability, with the dynamics of molecular and cellular mechanisms in order to establish that the environment of the caseous granulomas is hypoxic in humans. Using computation and simulated results, we demonstrate how hypoxia can occur in the human response to granuloma formation by considering oxygen levels, diffusion dynamics, and cellular interactions prevailing in the human lung. The dynamic genetic and metabolic adaptation of *Mtb* captured in our model helps explain how the pathogens biochemical response enables transition to long-term dormancy after initial infection, something that has not yet been demonstrated in existing models.

In the sections that follow we describe the model development process, including modeling of oxygen dynamics in the human lung, and the integration of the ABM and systems biology based model of *Mtb*. We present results of using our integrated model to simulate and study oxygen dynamics during tuberculosis infection and granuloma formation. In the final sections we conclude with a discussion of the mechanistic contribution of host physiological response to the development of hypoxia and the outcome of infection in human tuberculosis.

## Materials and methods

### Integrated multiscale model

**Figure 1:**
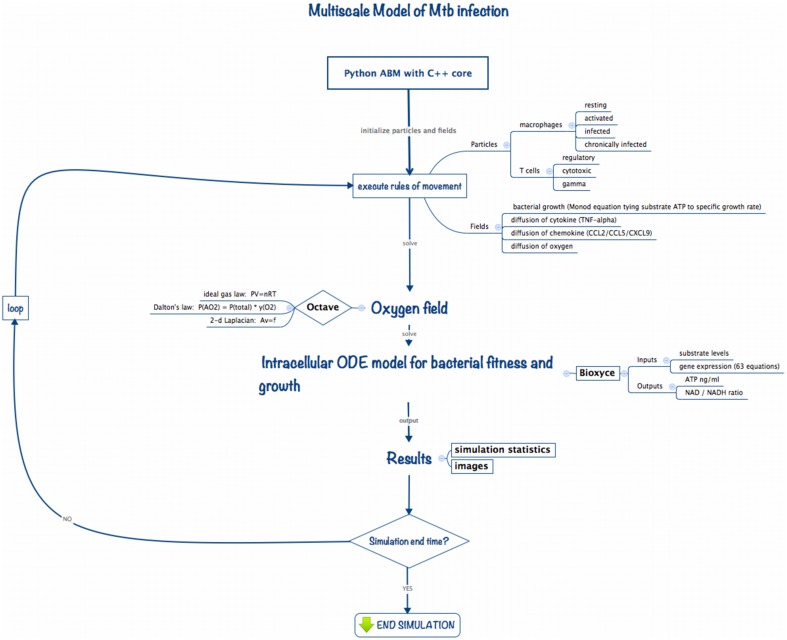
**Implementation schema for the integrated multiscale model of *Mtb* infection**.

Multiscale modeling has become increasingly necessary in computational biology in order to capture dynamics occurring at the diverse biological length and time scales germane to living systems. To study the impact of changes in the host physiological environment on *Mtb* persistence we developed ABM-PHYS, an integrated multiscale model of host physiological and immunological response to *Mtb* infection. *Schema of the ABM-PHYS Model.* Figure 1 outlines the structure and computational flow of the integrated ABM-PHYS model of TB granuloma formation. The model is comprised of three main components:

- ABM simulator developed using a Python-based software platform with C++ kernels to increase the speed of solving transient finite-difference diffusion equations;
- Octave routines to numerically solve the steady-state floating-point oxygen field;
- BioXyce, a systems biology modeling platform that solves a series of ODEs that determine substrate levels, overall microbial fitness and adenosine triphosphate (ATP) levels for the bacterial population in each ABM-PHYS grid cell (extracellular) or internally within a macrophage (intracellular). BioXyce enables parallel execution of *Mtb* systems biology models.

During an iteration of the model the ABM moves and updates cellular-scale particles (immune cells, bacterial populations), and recalculates cytokine and chemokine fields according to deterministic or probabilistic rules and analytical equations of state. The states of agents (host cells) in the model are updated, with each macrophage characterized based on its relative bacterial load and activation state as resting, infected, chronically infected or activated. The cell state can change due to interactions with environmental cytokines and chemokines, biological cells in neighboring grid cells, or due to the presence of bacteria in the macrophage's grid cell. These transitions are encoded as probabilistic or deterministic rules. The current state of a macrophage determines its immune response as well as its lifespan, which is tracked by an internal timer associated with each macrophage (and T cell).

We use an Octave-based routine (Eaton et al., 2014) to model and generate the steady state solution to the diffusion and uptake equation, and to calculate the dispersal for the updated oxygen concentrations on the cellular grid. Based on the oxygen levels in the extracellular environment or within the macrophage intracellular environment, the BioXyce-based systems biology model of bacterial response determines ATP levels and metabolic fitness variables (NAD/NADH ratio) for *Mtb*, which are used to calculate bacterial growth rates (via a Monod equation). This cycle is repeated to model the time evolution of the system. In the remainder of this section we describe the components of the model and how they interconnect to form the integrated ABM-PHYS multiscale model.

#### Agent-based model (ABM)

In order to incorporate spatiotemporal varying physiological and bacterial response into current *in silico* TB modeling methodologies we developed an integrable ABM platform. Using rules from published *in silico* models we implemented a standard TB ABM (ABM-ST) to verify the functionality of our Python-based simulation platform (Segovia-Juarez et al., 2004; Ray et al., 2009). While non-trivial and not the focus of this work, re-implementation of the standard TB ABM provided a comparative control for our *in silico* studies. We expanded on the standard TB ABM and designed a multiscale integrated model, ABM-PHYS, composed of an agent-based model to account for cellular-level host-pathogen interactions, a floating-point field model to represent spatiotemporal physiological changes in oxygen levels in the alveolar space, and multiple continuous valued fields to represent cytokine/chemokine gradients (TNF-α, CCL2, CCL5, CXCL9/10/11) in the extracellular space (Figure 1). We coupled our ABM to an ordinary differential equation (ODE) based dynamic systems biology model of *Mtb* metabolic pathways important in cellular respiration and energy (i.e., ATP) production, which we implemented using the BioXyce biological simulation platform (May and Schiek, 2009; May et al., 2013). (For notational distinction we will refer to the re-implemented standard agent based model of TB as ABM-ST and our extended model with the integrated physiological oxygen and *Mtb* systems biology models as ABM-PHYS. Both the re-implemented and our extended model are multiscale in nature.)

We developed our core ABM in the manner of Segovia-Juarez et al. (2004) and Ray et al. (2009) using the supplementary Materials and rules from the Ray et al. model (included in Ray et al., 2009 Supplement 1). As shown in **Table 2**, general parameters and parameter relationships for ABM-ST were implemented as reported in Ray et al. (2009) (Tables I–III, respective). We extended the ABM-ST framework to accommodate oxygen supply and consumption and included oxygen specific rules for cellular entities (see Appendices 1.2, 2, and 3 for new model rules implemented). We developed a host–pathogen interface module that translates grid-specific microenvironmental changes and *Mtb* metabolic state into pathogen specific gene regulation, thus enabling integration of the systems biology models of bacterial metabolism and growth [measured in colony forming units (CFUs)] with the ABM. The overall integrated, framework can thus be used as a multiscale model to capture the process of initial infection, host immune modulated physiological response, granuloma formation, and disease progression in a pulmonary tissue sample. The details of both the oxygen and intracellular extensions are described in the sub-sections that follow.

##### Modeling the extracellular microenvironment

The ABM treats two-dimensional (2D) space as a regular 2D grid. An individual grid point thus represents a small area or volume (in the case of 3D) of space, which can contain one or more cells of different types, e.g., macrophages, T-cells, bacteria. The two-dimensional computational model represents a 2 × 2 mm section of lung parenchyma, with 20 × 20 μm sized single grid cells (large enough to support the cell size of the largest agent, the macrophage) for a total of 10,000 grid cells. A grid cell may contain either 1 macrophage, a macrophage and a T cell, or two T cells, and up to 200 bacteria.

All of the parameters governing cell motion, the cell cycle (reproduction, infection, dying), and cell/cell interactions are encoded as heuristic rules with empirically-based parameters (see Tables 1–3). Diffusion equations capture the increase (production by either macrophages, T cells and bacteria), degradation (half-life), and diffusion of continuous-valued concentration species. Host cells secrete chemokines and cytokines depending on their state (e.g., CCL2, CCL5, CXCL9/10/11, and TNF-α), and bacteria secrete chemotactic factors or chemo-attractants that attract macrophages and T cells to their location. As cells in the model produce or consume chemokines and cytokines, the local concentration of species in their neighborhood affects movement of surrounding cells and attracts other cells to the site of infection or granuloma. There are sources and sinks for each species in the model, with the species represented as floating-point fields on the grid and their spatiotemporal diffusion equation defined as in Equation 1 below.

(1) $$\begin{array}{l} u_{t}=D\left[u_{xx}+u_{yy}\right]-g\left(x,y,t\right) \end{array}$$

**Table 1:** **Oxygen parameters used in the integrated multiscale ABM**.

**Parameter description**	**Default**	**Lower limit**	**Upper limit**	**Units**	**Distribution**	**Source**
Pulmonary blood volume (pulmonary.blood.source)	322	172	634	ml/*m*^2^/3.5 s	Uniform	Dock et al., 1961
Residual volume of air in lung (residual.volume)	1.697	1.0	2.75	Liters	Uniform	Nagelhout and Plaus, 2014
						Khurana, 2009
						Bartlett, 2010
Diffusion coefficient (in lung tissue—oxygen.difusion.coefficient)	3.08E−05	1.10E−04	4.00E−06	*cm*^2^/s	Uniform	MacDougall and McCabe, 1967
						Altman and Dittmer, 1974
						Hou et al., 2010
Maximum specific growth rate of extracellular *Mtb* (μ_*max*_ for Monod equation)	0.006	0.00095	0.06	Hourly	Uniform	Wayne and Hayes, 1996
Maximum specific death rate of *Mtb* (*K*_*d*_ for Monod equation)	0.0008	0.0001	0.0009	Hourly	Uniform	Wayne and Hayes, 1996
Maximum specific growth rate of intracellular *Mtb*	0.012	0.0019	0.12	Hourly	Uniform	Ray et al., 2009
Half velocity constant (K_*s*_ in Monod equation)	0.4227	Not varied		ng/ml		Wayne and Hayes, 1996
Consumption by resting macrophage (mr - resting.mac)	1.15	0.87	1.43	Micromoles/10^7^ cells/hr	Uniform	Conkling et al., 1982
Consumption by activated macrophage (ma)^*^	2.30	1.74	2.86	Micromoles/10^7^ cells/hr	Uniform	Loose, 1984
Consumption by infected macrophage (mi)^*^	3.45	2.62	4.28	Micromoles/10^7^ cells/hr	Uniform	Loose, 1984
Consumption by chronically infected macrophage (mc)^*^	4.60	3.49	5.71	Micromoles/10^7^ cells/hr	Uniform	Loose, 1984
Consumption by T cells	0.14375	0.10875	0.17875	Micromoles/10^7^ cells/hr	Uniform	
Consumption by *Mycobacterium tuberculosis* (O2.bact.consumption)^**^	20.80	10.00	35.00	*mm*^3^/hr/10^6^ bacteria	Uniform	Grieg and Hoogerheide, 1941
Alveolar surface area	130	118	142	*m*^2^	Not varied	Weibel, 1999

The oxygen parameters used to add the oxygen field to the ABM.

* Estimated from data concerning murine malaria.

** Estimated from data collected for E. coli.

**Table 2:** **Model parameters used for the standard and integrated ABM**.

**Parameter description**	**Default**	**Lower limit**	**Upper limit**	**Units**	**Distribution**	**Source**
Intracellular *Mtb* growth rate	0.002	0.0002	0.002	Per 10 min	Uniform	^*^
Extracellular *Mtb* doubling time	116	20	200	Hours	Log-Uniform	Ray et al. (2009)
Initial number of macrophages	105	Not varied				^*^
Probability of Mr killing bacteria (pK)	0.015	0.01	0.1	Per 10 min	Uniform	^*^
Probability of Mi activation by T cell (prob.actm)	0.05	0.0001	0.1	Per 10 min	Log-Uniform	^*^
Probability of macrophage recruitment (prob. recruit.mac)	0.05	0.01	0.1	Per 10 min	Uniform	^*^
Probability of T cell recruitment	0.075	0.01	0.1	Per 10 min	Uniform	^*^
Probability of T-γ cell	0.555	0.594	0.54	Per 10 min	Uniform	^*^
Probability of cytotoxic T cell	0.2775	0.297	0.27	Per 10 min	Uniform	^*^
Probability of a T cell moving onto an occupied compartment (Tmove)	0.01	0.00001	0.1	Per 10 min	Log-Uniform	^*^
Proportion of Treg cells out of all T cells recruited (T.prob.recruit.reg)	0.1	0.01	0.2	Per 10 min	Uniform	^*^
Chemokine diffusion rate (chemokine.diffusion.constant)	1.05E−07	1.67E−08	1.17E−07	*cm*^2^/sec	Uniform	^*^
Chemokine half-life (chemokine.halflife)	7.38E−01	6.0E−01	2.3E−00	Hours	Uniform	^*^
Combined TNF/chemokine threshold for T cell recruitment at a vascular source (r.T)	1.00E+03	1.00E+03	1.00E+05	Molecules	Log-Uniform	^*^
Combined TNF/chemokine threshold for Mr recruitment at a vascular source	1.00E+03	1.00E+03	1.00E+05	Molecules	Log-Uniform	^*^
CCL5 production rate	4.50E+05	6.00E+04	6.00E+05	Hours	Uniform	^*^
Macrophage CCL5 saturation threshold (CCL5uthresh)	1.41E+04	1.00E+04	1.00E+06	Molecules	Log-Uniform	^*^
Macrophage CCL5 threshold	2.00E+04	1.00E+04	1.00E+06	Molecules	Log-Uniform	^*^
TNF diffusion rate	1.09E−07	1.67E−08	1.17E−07	*cm*^2^/sec	Uniform	^*^
TNF half-life	3.6E−01	3.6E+01	11.55E+00	Hours	Uniform	^*^
TNF production rate	4.65E+06	6.00E+04	3.00E+07	Molecules Per hour	Log-Uniform	Marion et al. (2007)
Probability of TNF-induced apoptosis (p.apopt)	0.100	0.001	0.200	Per 10 min	Uniform^*^	^*^
Macrophage TNF detection threshold	7.00E+05	1.00E+05	1.50E+06	Molecules	Uniform^*^	^*^
Threshold Effect of TNF on Mr recruitment (tao.TNF.actm)	150	10	1000	Molecules	Log-Uniform	^*^
Carrying capacity for *Mtb* of a grid cell	220			Not varied		^*^
Macrophage lifetime	100	Not varied		Days		^*^
T cell lifetime	3	Not varied		Days		^*^
Maximum number of bacteria killed by resting macrophage	2	Not varied				^*^
Percent of internal bacteria being destroyed by killing	0.50	Not varied				^*^
No. of bacteria killed by activated macrophage	10	Not varied				^*^
Length of time T-reg incapacitates T-γ	110	Not varied		Minutes		^*^
Probability of cytotoxic T cell killing *Mtb* in mc death	0.75	Not varied				^*^
Probability cytotoxic T cells kills mc with bacterial release	0.20	Not varied				^*^

* Parameters are the same as those used in Ray et al. (2009) unless otherwise stated.

**Table 3:** **Summary of the Python modules developed for the multiscale simulator**.

**Python rules**	**Rule**	**Objective**
Simulator	simulator.py	Establish grid and neighborhood stencils; perform integrity checks
Particles	particle.py	Create new particles; initialize particles on grid
Macrophages:	resting.py	Phacytose bacteria or become infected; remove dead particles from grid (natural death or TNF-induced apoptosis)
	activated.py	Emit chemokine and cytokine; phagocytose bacteria; remove dead cells from grid (natural death or TNF-induced apoptosis)
	infected.py	Emit chemokine and cytokine; intracellular bacteria replication; remove dead particles for grid; may be activated by T cells
	chronic.py	Emit chemokine and cytokine; intracellular bacteria replication; bursts if max bacterial load reached; remove dead cells
	biasmove.py	Create particle with probability p in vascular source site if chemokine value is above threshold
	recruit.py	Remove dead T cells from grid
T cells:	Treg.py	regulates T-γ cell's ability to activate macrophages
	Tgamma.py	Probability of apoptosis on infected or chronically infected macrophage
	Tcytotoxic.py	Chance of perforin/granulysin-mediated killing of infected and chronically infected macrophages
	biasmove.py	If within threshold for at least one chemokine, make biased move in the direction of the highest concentration of chemo agent
	recruit.py	Create particle with probability p in vascular source site if chemokine value is above threshold
	death.py	Remove dead T cells from grid
Fields:	growth.py	Grow extracellular bacteria according to the Monod equation
	solve.py	Calculate the steady-state solution (Av = f) for the oxygen field via Octave routines
	gene expression.py	Calculate gene expression for the component genes and run BioXyce
	source.py	Add chemokine and cytokine (TNF/CCL2/CCL5/CXCL9) to chemotactic fields
	update.py	Run finite-difference diffusion routine
	apoptotic phagocytosis	Dictate macrophage behavior under hypoxia
Stats	stats.py	Time series report on key infection variables
Image	dump.py	Create images for animations

In Equation (1) *u*(*x, y, t*) is the concentration of the species at the grid point (*x, y*) at time *t*, *D* is the diffusion coefficient, and *g*(*x, y, t*) represents the source or sink function.

The diffusion equation is solved as a two-dimensional, second order, parabolic diffusion (heat) equation using an explicit finite difference method, which updates all the grid cells at each time step. For efficiency, this operation is performed with a custom C-routine called by the core ABM component, which is implemented in Python. Using the Courant stability criterion for solving diffusion equations on a grid of a given resolution, we chose the size of the time step for evolving the ABM-PHYS model based on the timescale required to track the most rapidly diffusing extracellular species in our model, which was oxygen. Rules that operate on a slower timescale (e.g., diffusion of large cells) are only invoked once every tens or hundreds of time steps. We set our time step to 4 s (average length of 1 breath) and updated the grid every 10 minutes of clock time, except for the oxygen field, which is solved every 18 h.

#### Modeling oxygen dynamics in the lung

We derived steady state oxygen levels for oxygen entering the system from two sources, the residual volume in the lung and the pulmonary blood volume, based on the ideal gas law:

(2) $$\begin{array}{l} PV=nRT \end{array}$$

where *P* and *V* represent pressure and volume, *n, R*, and *T* represent moles of oxygen, ideal gas constant, and temperature, respective. Using the ideal gas law we calculate the amount of oxygen available at the boundary (source) cells and each sink cell on the grid. Source cells are either grid cells located on the periphery (residual oxygen in the lung) or randomly distributed within the interior of the grid (pulmonary blood volume). Sink cells are grid cells where macrophage, extracellular bacteria, or T cells reside. A sample calculation is presented in Appendix 2 in Supplementary Material (See Sershen et al., 2014 Section Materials and Methods for an extended discussion of the implementation of the oxygen field).

We compute the drop in partial pressure of oxygen for the 2 × 2 mm parenchymal section in order to calculate oxygen levels and determine the occurrence of hypoxia during the course of our simulation model. We assume that the overall partial pressure change in the lung alveoli is negligible. This means that overall the pressure remains at the rate of a healthy male (≈ 99.7–105 mmHg) but the drop in partial pressure over the individual grid cells and granuloma aggregates are computed. We also assume that the drop in the partial pressure of oxygen in the alveoli (*P*_*AO*2_) is directly proportional to the mole fraction of oxygen in alveolar tissue (*y*_*O*2_), as per Dalton's law of partial pressure:

(3) $$\begin{array}{l} P_{AO2}=P_{total}*y_{O2} \end{array}$$

We use the computed mole fraction of oxygen in alveolar tissue (*y*_*O*2_) to determine the amount of O2 flowing in from source grid cells in our model.

We implement oxygen diffusion within the grid as a floating-point field in our ABM-PHYS model in the manner described in Sershen et al. (2014). The spatial and time dependence of the concentration is represented by the diffusion equation (Equation 1) with Dirichlet boundary conditions. In our model, grid cells that contain macrophages, T cells or bacteria that consume oxygen are sinks. Boundary grid cells, which receive an influx of oxygen from adjacent tissue, are sources. Because oxygen diffuses quickly (*D*≈3.08*e*−5 in lung tissue Hou et al., 2010), accurately tracking transient variations in oxygen concentration would require a time step equal to 0.032 s to satisfy the Courant–Friedrichs–Lewy (CFL) condition for an explicit finite-difference method (Sershen et al., 2014). However, for our model we are mainly interested in the quasi-static oxygen concentration profile, which assumes the concentration quickly comes to equilibrium with any change in the sources and sinks. Therefore, we consider steady-state levels of oxygen in and through the lung parenchyma derived from the net amount left in tissue deposits due to respiration and the amount of oxygen available from pulmonary blood volume (see Table 1). Given that the overall change in spatial distribution of O2 sources and sinks is relatively slow compared to the rate of oxygen diffusion, as the ABM evolves we solve and update the oxygen concentration profile once every 18 h, which is a much longer timescale than the timescale for diffusion. Numerically, this is accomplished by dropping the time-dependent left-hand-side of the diffusion equation (Equation 1) and solving the resulting matrix equation D^*^Au = *g*, where *A* is a sparse matrix representing the connectivity of the grid cells (five point stencil in 2D), *u* is the concentration vector (one unknown for each of the 100^2^ grid cells), and *g* is the source/sink vector for each grid cell. The matrix equation can be solved efficiently using iterative conjugate gradient methods (Calvert, 2014), available in GNU Octave.

Oxygen tension or the partial pressure of oxygen in the blood in well-irrigated human parenchymal tissue is generally between and 14% (30–106 mmHg; Iovanic, 2009) of atmospheric pressure, In hypoxic tissue the oxygen tension is generally below 2% (15 mmHg; Lewis et al., 1999). We characterized hypoxia in our simulated ABM-PHYS granuloma as oxygen tension less than two percent. We could thus determine whether a granuloma contained hypoxic areas and/or anoxic areas (0 % oxygen). The oxygen parameters in Table 1 represent the steady state oxygen levels for a range of air intake values (12–20 breaths per minute; Silverthorn, 2013). These were varied within the biological ranges shown in Table 1 in the sensitivity analysis.

##### Expanding the cellular response models to account for oxygen dynamics

Consumption of oxygen is based on the number and type of macrophages, T cells and bacteria present within a grid cell. Oxygen consumption values for bacteria are based on data from *E. coli* (Grieg and Hoogerheide, 1941). Activated and infected macrophages consume more oxygen than resting macrophages, with infected macrophages consuming up to 12 times more oxygen (Loose, 1984). To approximate state-dependent oxygen consumption rates for macrophages in our model, we assume that activated macrophages consume twice that of resting macrophages, infected macrophages consume three times more oxygen than resting macrophages and that chronically infected macrophages consume four times more oxygen than resting macrophages. Since T cells are approximately 1/8 the volume of the macrophage, they consume 1/8 the amount of oxygen of resting macrophages.

We added rule-based mechanisms to reflect macrophage response to oxygen dynamics in the host environment and within containment granulomas. Hypoxia affects the activity and function of host cells in areas such as morphology, expression of cell surface markers, cell survival, phagocytosis, metabolic activity, and production of nitric oxide, as well as cytokine secretion. In healthy tissues, oxygen tension is usually between 2.5 and 9 % (20–70 mmHg; Lewis et al., 1999), therefore in the model we define hypoxia as oxygen tension <2 % (15 mmHg), which is double the level of dissolved oxygen in the Wayne model's NRP stage 1 (1 % oxygen; Wayne and Hayes, 1996). Hypoxia/anoxia has been shown *in vitro* to reduce cell viability by about 20 percent in rat and murine macrophages (Lewis et al., 1999). Human macrophages under low oxygen conditions switch from oxidative phosphorylation to anaerobic glycolysis, Simon et al. (1977); Roiniotis et al. (2009) with the outcome being that cells adapt to low O2 conditions and very little cell death occurs due to hypoxia Lewis et al. (1999). Accordingly, we allowed for a small probability (*p* < 0.001) of macrophage apoptosis under enduring hypoxic or anoxic conditions; see Appendix 1 in Supplementary Material for details.

Another function that is affected by low oxygen conditions is phagocytosis. In short duration (6 h or less) hypoxia tends to degrade the ability of macrophages to phagocytose viral particles (Leeper-Woodford and Mills, 1992). But upon adaptation of the alveolar macrophage and onset of anaerobic glycolysis, phagocytosis increases two-fold in mice under enduring hypoxic conditions (up to 96 h; Lewis et al., 1999), but decreased in rabbits. Since there was not a clear consensus regarding how hypoxia affects phagocytosis, this feature was not implemented in the model.

Hypoxia also stimulates cytokine secretion by macrophages, in particular TNF-α production (Lewis et al., 1999). To capture this effect TNF-α secretion was doubled for macrophages under hypoxia. Though Lewis et. al did not specify the exact amount of TNF-*alpha* induction, they did note that other cytokines, such as prostaglandin E2 (PGE2) doubled secretion under hypoxic conditions.

Alveolar macrophages also experience changes in metabolic activity and production of nitric oxide in the presence of low oxygen tension as a result of hypoxia-mediated modulation of gene regulation and the binding of the transcription factor hypoxia-inducible-factor-1 (HIF-1). These dynamics were not included but can be addressed by additional systems biological models for macrophages response to hypoxia in future versions of the model.

#### Metabolic model of *mtb* adaptation and growth

Using an *in vitro* model of *Mtb* during active growth and persistence, Wayne and Hayes (1996) demonstrated that when the rate of oxygen depletion is sufficiently slow, *Mtb* metabolically adjusts to the lack of oxygen by shifting through two stages of non-replicating persistence (NRP1 and NRP2). Conversely under growth conditions that result in high rates of oxygen depletion, *Mtb* failed to persist presumably due to the inability of the mycobacterium to metabolically adapt to the rapid microenvironmental change. We use a systems biology based metabolic model of *Mtb* metabolism to link the physiological effect of host immune response, namely modulation of physiological oxygen gradients, to pathogen adaptation and persistence (May et al., 2013). We model biochemical pathways involved in *Mtb* oxygen-dependent energy production and the recycling of key metabolic co-factors under varying oxygen conditions for both intracellular (bacteria within macrophages) and extracellular bacteria (bacteria outside of macrophages but within the lung parenchyma). Since oxygen is critical to ETC function and ATP production, we expect physiologically low oxygen levels to reduce bacterial ATP production, which then would foster a decline in the metabolic fitness of *Mtb* populations and a consequential reduction in bacterial load. However, the Wayne NRP model suggests that the dynamics of oxygen depletion as opposed to simple bioavailability is an important determinant to pathogen elimination vs. persistence, therefore the dynamics of the physiological host response will also contribute to *Mtb* elimination.

Using our existing model of *Mtb* metabolic response to low oxygen and small molecule inhibition (May et al., 2013), we expanded the model to include a more mechanistic representation of the electron transport chain. Our metabolic model of mycobacteria includes the TCA cycle, glyoxylate bypass, glyoxylate-to-glycine shunt, electron transport chain, and oxidative phosphorylation and was derived from published theoretical models and empirical descriptions of the biochemical networks (Table 4; Wayne and Hayes, 1996; Singh and Ghosh, 2006; Beste et al., 2007; Fisher et al., 2009; May et al., 2013). The model implicitly takes into account the role of menaquinone/menaquinol in the production of the proton motive force, however we do not explicitly track menaquinone levels in the model. We account for cellular growth through a simplified ATP dependent biomass production reaction (47 ATP = 1 BIOMASS). The *Mtb* reaction rate equations were generated using kinetic parameters from the BRENDA Enzyme database, empirical data on *Mtb* growth under low oxygen conditions, and a Michaelis–Menten reaction kinetics framework with initial enzyme concentrations nominally set to values up to two orders of magnitude less than initial substrate levels (Wayne and Hayes, 1996; Smith et al., 2003; Nelson and Cox, 2005; Scheer et al., 2011).

**Table 4:** **Reactions included in the *Mtb* systems biology model to capture the TCA cycle, glyoxylate to glycine shunt, electron transport chain, and oxidative phosphorylation**.

**Enzyme name**	**Reaction**	**Genes**
Citrate synthase (CS)	1 OA + 1 ACCOA = 1 CIT + 1 COA	Rv0896 OR Rv0889c OR Rv1131
Aconitase (ACN)	1 CIT = 1 ICIT	Rv1475c
Isocitrate dehydrogenase 1 (ICD1)	1 ICIT = 1 AKG	Rv3339c OR Rv0066c
Isocitrate dehydrogenase 2 (ICD2)	1 ICIT = 1 AKG	Rv3339c OR Rv0066c
Alpha-ketoglutarate decarboxylase (KGD)	1 AKG = 1 SUCCSAL	Rv1248c OR Rv0555
Succinic semialdehyde dehydrogenase (SSADH)	1 SUCCSAL = 1 SUCC	Rv0234c OR Rv1731
Succinate dehydrogenase (SDH)	1 SUCC + 1 FAD = 1 FUM + 1 FADH2	Rv3318 AND Rv3319 AND Rv3316 AND Rv3317
Fumarase (FUM)	1 FUM = 1 MAL	Rv1098c
Malate dehydrogenase (MDH)	1 MAL + 1 NAD = 1 OA + 1 NADH	Rv1240
Isocitrate lyase 1 (ICL1)	1ICIT = 1GLX+1SUCC	Rv0467 OR (Rv1915 AND Rv1916)
Isocitrate lyase 2 (ICL2)	1ICIT = 1GLX+1SUCC	Rv0467 OR (Rv1915 AND Rv1916)
Malate synthase (MS)	1 GLX + 1 ACCOA = 1 MAL + 1 COA	Rv1837c
Alanine dehydrogenase/glycine dehydrogenase (GDH/ALD)	1 GLX + 1 NADH = 1 GLY + 1 NAD	Rv2780 OR GDH
NADH dehydrogenase (NUO)	NADH + 0.5 O2 = NAD + 2H	Rv3145 AND Rv3146 AND Rv3147 AND Rv3148 AND Rv3149 AND
		Rv3150 AND Rv3151 AND Rv3152 AND Rv3153 AND Rv3154
		AND Rv3155 AND Rv3156 AND Rv3157 AND Rv3158
NADH reductase (Non-proton translocating, NDH)	1 NADH + 0.5 O2 = 1 NAD	Rv1854c OR Rv0392c
Succinate dehydrogenase (SDH)	FADH2 + 0.5 O2 = FAD + 2H	(Rv3318 AND Rv3319 AND Rv3316 AND Rv3317) OR
		(Rv1552 AND Rv1553 AND Rv1554 AND Rv1555)
ATP Synthase (ATPase)	1 ADP + 1 PI + 4 H = 1 ATP	Rv1308 AND Rv1304 AND Rv1311 AND Rv1310 AND Rv1305
		AND Rv1306 AND Rv1309 AND Rv1307

To relate the enzyme concentration used in the *Mtb* metabolic model to empirically observed growth and oxygen related fold changes, we multiply reaction velocity by the relative fold change value of genes associated with the production of the enzyme. Gene to enzyme correlations are based on the Beste et al. metabolic network model of *Mtb* (Beste et al., 2007). Using a fractional occupancy approach, the effective reaction rate is represented as a function of gene expression: *v*_*Effective*_ = Y^*^v, with Y = *active*/(*sum all forms*), where active indicates the activating form of the gene or gene complex needed to produce the enzyme and the denominator consists of all genetic forms associated with the enzyme (Sauro, 2012). We use empirical data from *in vitro* studies of *Mtb* NRP to develop a theoretical approximation for gene expression (Wayne and Hayes, 1996; Voskuil et al., 2004).

##### Dynamic control of gene expression

To predict gene expression levels in our metabolic model of *Mtb* we used experimental data from the Wayne and Hayes *in vitro* NRP study (Wayne and Hayes, 1996) and supplementary data from Voskuil, et al.'s study of gene expression during *Mtb* hypoxia induced NRP (Voskuil et al., 2004). We fit both aerobic and hypoxic (slow-stirred) gene expression data from Voskuil et al. (2004) to a statistical model of gene regulation using percent oxygen consumption and ATP production values from Wayne and Hayes (1996). For these two independent measures, we found the polynomial curves of best fit in order to extract data at consistent time points for the two oxygen-dependent scenarios. We correlate four independent variables: change in time, change in percent oxygen consumed, depletion rates for oxygen, and ATP production to the gene expression levels for each of the 59 genes that are components of enzymes active in the ETC cycle (see Table 4, third column for list of genes). By using change in time and change in percent oxygen instead of explicit time and percent oxygen, we were able to correlate the rate of gene expression to the relative dynamics of the microenvironment rather than a fixed chronological time frame. We used a combination of linear and non-linear fits to construct individual multiple regression models for each of the 59 genes of interest. The model was trained using gene expression data from the 80 day interval in the Voskuil et al. study (Voskuil et al., 2004), which reports data up to 60 days for the aerated model and 80 days for the slow-stirred/NRP model. We combined the aerated and NRP data sets to generate a single 80-day data set representing *Mtb* response to two different environmental conditions. The combined observation dataset was used to generate a separate regression equation for each gene model. While there was not a unique empirical dataset available for cross-validation of the model, we validated the gene model using the non-combined data for the fully aerated or the hypoxic slow-stirred condition (see Supplementary Figure 1 for profiles of two of the genes modeled). The predicted gene expression trajectories were in agreement with the actual data from the training interval; *R*-square values were in the range of 0.80–0.99 and regression F statistics were generally < 0.01 for the 59 genes included in our model. The resulting regression-based models are used to predict gene expression levels for genes corresponding to enzymes in the *Mtb* metabolic model.

##### Bacterial growth model

To model ATP-dependent bacterial growth and death, we used the Monod equation:

(4) $$\begin{array}{l} \mu =\frac{\mu max*ATP}{K_{s}+ATP}-K_{d}*\left(1-normalized\left(\frac{NAD}{NADH}\right)\right)*ATP \end{array}$$

where μ is the specific growth rate under the current microenvironmental conditions, μ_*max*_ is the maximum specific growth rate, K_*s*_ is the concentration of ATP corresponding to the half growth rate constant, K_*d*_ is the death rate, NAD is the oxidized form of nicotinamide adenine dinucleotide and NADH is the reduced form of the coenzyme. Once the growth rate is determined via the Monod equation, extracellular bacterial growth is modeled according to the ODE:

(5) $$\begin{array}{l} B_{E}\left(t+1\right)=B_{E}\left(t\right)+\alpha_{BE}*B_{E}\left(t\right)*\left(1-\left(B_{E}\left(t\right)∕\left(K_{BE}*1.1\right)\right)\right) \end{array}$$

where α_*BE*_ is set to the value of μ determined using the Monod Equation. α_*BI*_ is a multiple (1.5–2X) of α_*BE*_ (Segovia-Juarez et al., 2004). Growth rates of intracellular bacteria are generally higher than that of extracellular bacteria (Zhang et al., 1998; Segovia-Juarez et al., 2004). Accordingly we set the maximum base intracellular growth rates to 1.5–2 times higher than their extracellular counterparts. Intracellular bacteria grow according to the ODE model (Segovia-Juarez et al., 2004):

(6) $$\begin{array}{l} B_{I}\left(t+1\right)=B_{I}\left(t\right)+\alpha_{BI}*B_{I}\left(t\right) \end{array}$$

The growth rate equation is fit to the data from Wayne and Hayes (1996) to relate growth rate to the level of bacterial ATP in the system and normalized to a value between 0 and 1. The death rate is modulated by the metabolic fitness of the bacteria as measured by the $\frac{NAD}{NADH}$ substrate ratio based on values given by the intracellular systems biology model. The number of extracellular bacteria in one grid cell is bounded by the carrying capacity of the occupied grid cell. When a grid cell reaches capacity, the excess bacteria are distributed to neighboring cells.

The gene expression and the metabolic models of *Mtb* adaptation and growth were integrated into the multiscale model using the algorithm outlined in Appendix 3 in Supplementary Material. Using our ABM-PHYS model, we study how the host cellular immune response can dynamically modulate physiological oxygen levels and investigate the contribution of oxygen dynamics to the three possible infection outcomes (clearance, containment, and dissemination).

### Uncertainty quantification, model integration, and model validation

Using a modular approach, we optimized and validated the *Mtb* systems biology model implemented using BioXyce and then performed uncertainty quantification on the integrated multiscale model. The metabolic model was calibrated using growth and ATP data presented in the Wayne and Hayes study for the aerated condition (Wayne and Hayes, 1996). In addition to constraining the model to positive ATP values, NAD:NADH and FAD:FADH ratios were constrained to correspond to ratios observed for wildtype *Mtb* (Singh et al., 2009). We used the DAKOTA (Adams et al., 2010) software to formulate a simple genetic algorithm that identified globally optimal *Mtb* metabolic model parameters that fit the experimental ATP data trajectories to accuracy within 1e-04.

We coupled the python-based host ABM platform to the BioXyce platform (May and Schiek, 2009; May, 2011) by passing spatiotemporally varying oxygen levels and gene expression values calculated by the ABM to the BioXyce *Mtb* model. Using key metabolite concentrations (NAD, NADH, ATP) returned by the BioXyce simulation the host ABM calculates the extracellular and intracellular bacterial growth rates at each simulation time point in the multiscale model (see Appendix 3 in Supplementary Material for additional integration rules). We implemented a parallel LHS-based sensitivity analysis of our integrated model using the DAKOTA toolkit (Marino et al., 2008; Adams et al., 2010). We treat our model as a black box (a sink for varied parameter inputs and source of outputs for variables of interest), and developed a Perl wrapper to parallelize the model analysis, thus minimizing the simulation time required for running multiple model replicates concurrently. We performed sensitivity analysis to investigate the two sources of randomness in our multiscale model: aleatory (stochastic) and episystemic uncertainty. To minimize the impact of aleatory uncertainty, we ran three simulations each with the same LHS parameters but with different random number seeds and averaged the results. To identify episystemic uncertainty, we performed LHS over the biological range for each parameter, generating *N* = 300 individual sample runs which were averaged to produce a total of 100 multi-sample average runs. Among other analytical measures, DAKOTA returns matrices of partial correlation coefficients (PCC) and partial ranked correlation coefficients (PRCC), which we used to identify statistically significant model drivers. PCC measures the degree of linear correlation between the output and input variables. The PRCC measures the degree of correlation between the input and output variables provided that the relationship between both is monotonic (may also be non-linear).

We categorized the phenotypic outcome of each sample run as a clearance, containment (granuloma formed), or dissemination outcome using the algorithm described in Appendix 1 in Supplementary Material. Treating the sample outcomes as a representative population, we validated our integrated model by comparing the percentages associated with the relative number of samples in each qualitative outcome to *in vivo* data from primate studies conducted by Gideon et al. (2015) and epidemiological data from the WHO on the rates of latent TB disease (represented as containment; World Health Organization, 2013). We used Chi-square tests for normal data and the non-parametric K–S test to compare distributions derived from *in vitro* and *in vivo* experiments with our *in silico* distributions using bacterial loads as an indicator of disease outcomes. Using data from the sensitivity analysis, we evaluated how closely the distribution of lesions resulting from simulated *Mtb* infection emulated the latent infection outcome. In the section that follows we present results of individual simulations and results that represent an average of multiple simulations as outlined in Table 7.

## Results

### Simulated host–pathogen interactions and the role of oxygen in structural immune response

**Figure 2:**
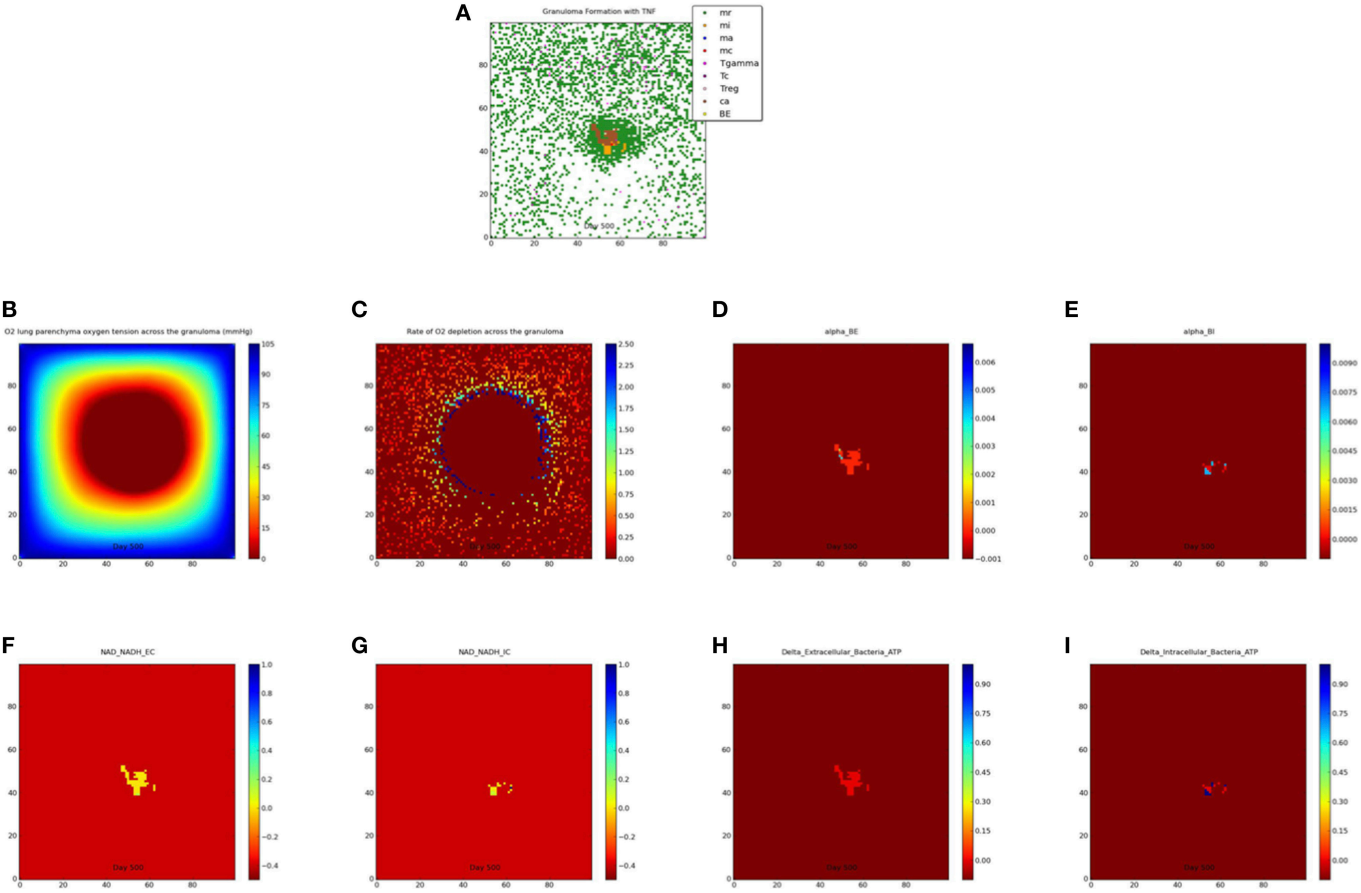
**Containment granuloma with TNFα, contains bacteria characterizable as in a state comparable to non-replicating persistence at 500 days**. **(A)** Partial pressure of oxygen across the granuloma **(B)** oxygen depletion rate **(C)** bacterial growth rates for extracellular **(D)** and intracellular **(E)** bacteria; scaled NAD/NADH ratio for both extracellular **(F)** and intracellular **(G)** bacteria; change in ATP concentration for extracellular **(H)** and intracellular **(I)** bacteria.

We simulate the outcome of an infected macrophage in a host with normal pulmonary capacity. Figure 2 shows a simulation that resulted in a containment granuloma with most of the bacteria in a state of NRP at 500 days post-infection. As stated previously, containment reflects a state of equilibrium in which the rate of host immune cell recruitment, activation, death and renewal are balanced by *Mtb* growth, death, and metabolic adaptation to the physiological microenvironment, resulting in the maintenance of the *Mtb* infection in a latent or persistent state. In our *in silico* ABM-PHYS studies, in addition to clearance, containment, and dissemination outcomes we often encountered a fourth category of scenarios where *Mtb* is transiently contained in granulomas at 200 days post-infection but the granuloma either clears the infection or the bacteria disseminates by 500 days post-infection. We named this category ”transient containment,” and mathematically defined transient containment according to the behavior of the derivatives of external bacteria and resting macrophage recruitment. (See Appendix 1 in Supplementary Material for detailed algorithm used to separate the qualitative outcomes, including transient containment.) We simulated up to 500 days post-infection to capture the true containment scenario. However, simulating up to 500 days required substantial computational time, therefore in our sensitivity analysis studies and results we show the averages from 200-day simulations given that the majority of outcomes are determined by the 200 day simulation time step. Figure 2A represents a 2 × 2 mm section of the lung parenchyma where the host cell and *Mtb* interactions lead to granuloma formation. Following the representation convention of previously published TB ABMs (Segovia-Juarez et al., 2004; Marion et al., 2007; Ray et al., 2009), which facilitates comparative analysis, in the legend mr, mi, ma, mc refer to resting, infected, activated and chronically infected macrophages, respectively; T-γ, Tc, and Treg refer to T-γ cells, cytotoxic T cells and regulatory T cells, respectively. Note in Figure 2A the extensive caseous regions within the core of the granuloma, caused by repeated bursting of macrophages during the infection cycle. A grid cell is designated as caseous if six or more macrophages burst within the grid cell over the course of the simulation; see for example Ray et al. (2009). Figure 2B shows the partial pressure of oxygen in units of mmHg across the cellular grid, with oxygen diffusing into the center from the boundary grid cells. Figure 2C shows the rate of oxygen depletion across the granuloma, which is calculated as the difference in oxygen levels over time. As shown in Figure 2C the highest oxygen depletion rate is within the inner region of the granuloma, presumably due to host cells infiltrating the site of infection. The center has very low depletion levels, as does the outer regions of the grid. However, while the center is hypoxic and possibly anoxic (Figure 2B and caseous regions in Figure 2A), the outer regions are well oxygenated.

The host cellular response and corresponding physiological changes in environmental oxygenation modulates the genetic response of *Mtb* as exhibited in Supplementary Figure 2, which shows an example of predicted fold change based on oxygen availability and days post-infection for two of the 59 genes modeled. The fold change for dissemination and containment in the simulations mirror the Voskuil et al. profiles for aerobic and hypoxic/NRP conditions (inset upper right). The predicted change in gene expression corresponds to changes in enzyme levels and consequentially in reaction dynamics for the *Mtb* metabolic network. Supplementary Figure 3 shows the gene expression-driven variation in metabolite levels for extracellular bacteria over 200 days post-infection for clearance, containment, transient containment and dissemination (Supplementary Figures 3A glycine; **3B** malate; **3C** isocitrate; **3D** glyoxylate).

Figures 2D,E show the growth rates of extracellular and intracellular bacteria respectively at 500 days post-infection, which is modulated by the systems biology model of *Mtb* metabolism. Extracellular growth rates are almost entirely zero and intracellular growth rates are predominately zero. Non-zero intracellular growth rates occur where residual oxygen carried in macrophages recruited from well-oxygenated areas enables growth of internal bacteria. Zero growth rate areas for extracellular bacteria correspond mainly to caseous regions (Figures 2A,D). These observations suggest that a large number of the bacteria in the granuloma in Figure 2A may be characterized as in a state of NRP based on rate of growth. Given that the systems biology model of *Mtb* metabolic response is the same for extracellularly and intracellularly located bacteria, the host-mediated changes in the physiological environment is the variable that drives the observed difference in metabolic output of the bacteria. Therefore, the location-specific environment of the bacteria contributes to the emergent metabolic characteristics of intracellular vs. extracellular bacteria (Figures 2D–I).

Figures 2F,G portray the relative extracellular and intracellular NAD/NADH ratios respectively, which are used as a fitness measure to modulate the death term in the Monod equation. Several NAD/NADH ratios for both extracellular and intracellular bacterial populations are close to zero with some populations, particularly intracellular *Mtb* populations, close to one (the maximum relative fitness value). While difficult to visualize in Figures 2F,G there are some populations of intracellular bacteria with higher NAD/NADH levels, which is supported by the slightly higher average NAD/NADH ratio observed for intracellular bacteria (compare Supplementary Figures 4E and Supplementary Figures 4F) after 100 days of simulation for the containment scenario. Negative values in these plots represent the background matrix and have no physiological interpretation. Figures 2H,I illustrate the change in ATP levels for both intracellular and extracellular bacteria. The ATP plots show more intracellular bacteria populations with high delta*_*ATP (change in ATP over time) than the extracellular bacteria. The intracellular regions with greater ATP dynamics correspond to intracellular regions with relatively higher growth rates (Figure 2E, alpha*_*BI). This observation suggests that intracellular bacteria may have relatively higher metabolic activity than extracellular bacteria for the containment scenario, as supported by the average NAD/NADH values in Supplementary Figures 4E,F. However, in the containment scenario the average ATP concentrations (Supplementary Figures 4G,H) for extracellular bacteria are slightly more than two times that of intracellular bacteria, but this does not correlate to a higher extracellular growth rate when compared to intracellular growth rates (Figures 2D,E, Supplementary Figures 4C,D). Given that ATP is a major determinant in bacterial growth and that growth rate also indirectly accounts for bacteria removed by the host, we postulate that the additional reduction in extracellular bacterial growth rate is due to host-mediated uptake and killing of extracellular Mtb. As designated for the NAD/NADH plots, background matrix assumes a negative value so that the zero levels may be distinguished from background.

### Comparative analysis of model outcomes

#### Comparison of *in silico* models of infection

**Figure 3:**
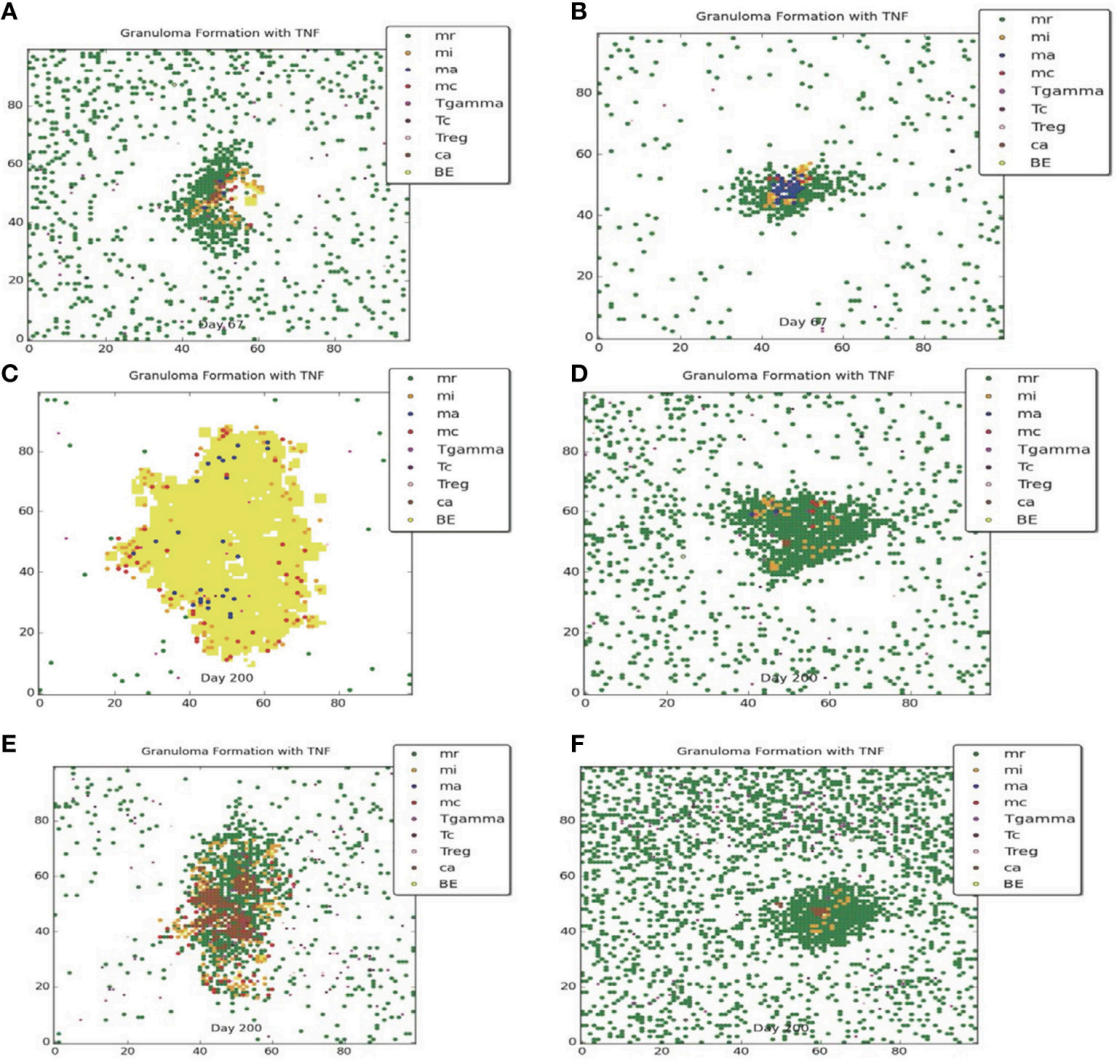
**Comparison of transient containment outcomes at 67 days post infection (A,B) and 200 days post infection; the standard re-implemented model (ABM-ST) results in dissemination (C) and the physiologically-based model (ABM-PHYS) results in a loosely packed containment (D)**. True containment outcomes shown at 200 days post-infection (both ABM models result in containment **E,F**). Simulation models use the same parameters for ABM-ST (left) and ABM-PHYS (right). See Appendix 4 in Supplementary Material for relevant parameters.

**Figure 4:**
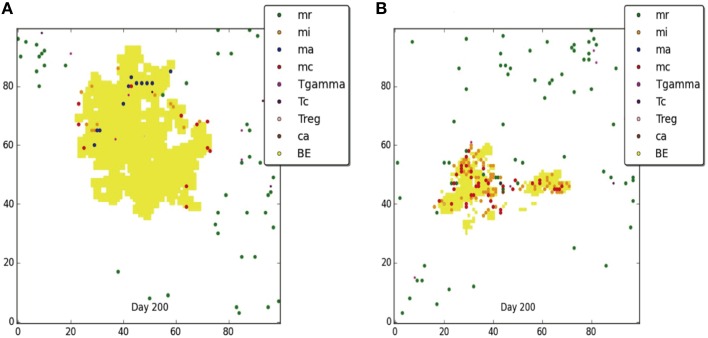
**Comparison of dissemination outcomes in the absence of TNF-α for ABM-ST (A) and ABM-PHYS (B)**. See Appendix 4 in Supplementary Material for relevant parameters.

Figures 3, 4 compare containment and dissemination outcomes, respective, for ABM-ST (left figures) and ABM-PHYS (right figures). These figures are generated using the same parameter sets fixed for each qualitative outcome model [Figures 3A–F; see Appendix 4 (Supplementary Table 1) in Supplementary Material, for parameters used for the two containment scenarios] and the dissemination scenario (Figures 4A,B). Using model parameters that correspond to containment outcomes, we compare simulations that result in a well formed, compact containment granuloma (Figure 3F) vs. a less tightly packed transient containment granuloma in the ABM-PHYS model (Figure 3D). The probability of macrophage recruitment from a vascular source was set at a power of ten lower in Figures 3A–D than Figures 3E,F (0.006 vs. 0.075) and the number of TNF-α molecules secreted hourly was slightly lower for Figures 3A–D than Figures 3E,F (3.15e+06 vs. 4.35E+06). All other parameters remained the same for the two containment scenarios [Appendix 4 (Supplementary Table 1) in Supplementary Material]. There were no parametric differences between ABM-PHYS and the ABM-ST model, only differences due to the modeling methodology. Specific changes in the structure of the ABM-PHYS model include: addition of the oxygen field and immune response mediated modulation of physiological oxygen, associated oxygen-driven host response, and the use of fixed growth rates for bacteria in the ABM-ST model vs. the use of bacterial growth rates modulated by *Mtb* metabolic adaptation in the ABM-PHYS simulation model. In comparing the two models we first qualitatively evaluate the effect of oxygen dynamics on the organization, structure, and function of the granuloma. We also quantitatively compare their performance using the metric of extracellular bacterial load as in Segovia-Juarez et al. (2004) and Ray et al. (2009).

Both the ABM-ST and ABM-PHYS initially control and contain *Mtb* as evidenced by the day 67 results (Figures 3A,B). However, ABM-ST eventually results in dissemination whereas the ABM-PHYS model results in a containment outcome with low amounts of extracellular and intracellular bacteria at 200 days (compare Figures 3C,D). The difference in outcomes is likely due to the non-dynamic growth rate of bacteria in the ABM-ST model, which is at the maximum level and not modulated by fluctuating oxygen conditions resulting from the physiological immune response considered in the ABM-PHYS model. The static bacterial growth rate leads to ABM-ST model skewing toward dissemination with a reduced number of containment outcomes, compared to the ABM-PHYS model which results in more containment outcomes (outcome percentages shown in **Figure 6C**). Presented in Figure 3E (ABM-ST) and Figure 3F (ABM-PHYS) are containment scenarios with the same parameters for the two models [see Appendix 4 (Supplementary Table 1) in Supplementary Material, for associated parameters]. The ABM-ST model results in a loosely packed granuloma with extensive caseous regions. The granuloma produced by the ABM-PHYS model is tightly packed, and features hypoxic or nearly hypoxic regions (data not shown). Again in part due to the constant maximum growth rate of intracellular bacteria there is an increased likelihood of macrophage bursting, leading to more caseous regions. The ABM-PHYS model also features caseous regions, but these are smaller and more compact, which is likely due to the modulated growth rate driven by changes in physiological oxygen levels and the bacterial model.

Using the dissemination parameters listed for the dissemination model in Appendix 4 (Supplementary Table 1) in Supplementary Material, we compared dissemination outcomes for ABM-ST vs. ABM-PHYS model. Given the tendency of the ABM-PHYS model to skew toward containment (**Figure 6C**), we significantly reduced TNFα in the system to generate a more comparable dissemination scenario for both models. Based on qualitative observations for the dissemination model (Figures 4A,B) the ABM-ST model results in more pronounced bacterial growth and dispersion during dissemination. However, accounting for physiological factors in the ABM-PHYS model contributes to a less dispersed dissemination outcome with more caseous regions and a comparatively reduced extracellular bacterial load.

**Figure 5:**
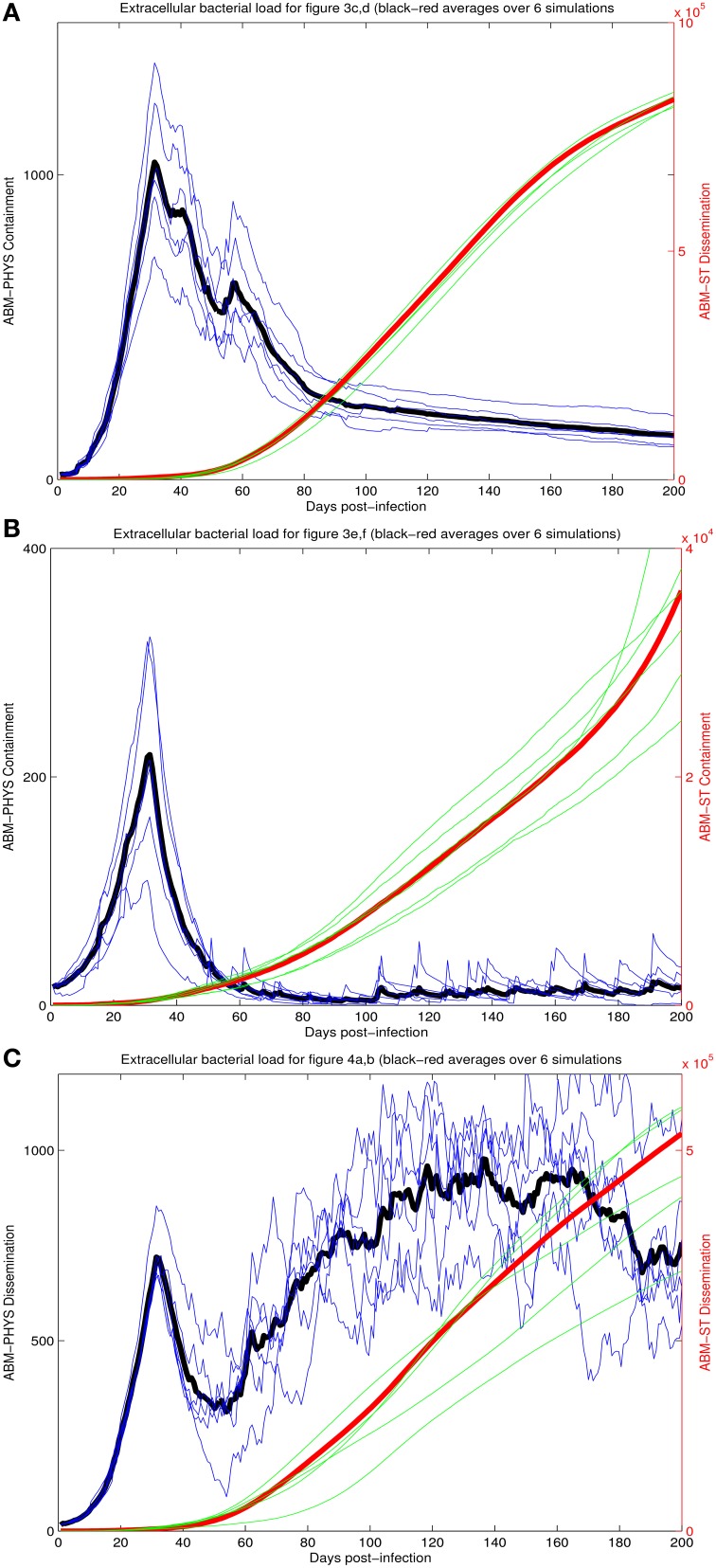
**Comparison of extracellular bacterial load of ABM-ST vs. ABM-PHYS model for each of the three scenarios in Figure 3: transient containment (A, Figures 3A–D); containment (B, Figures 3E,F) and dissemination (C, Figures 3G,H)**. Results represent an average of six simulations in all scenarios except the transient containment scenario for ABM-ST, which has five simulations. Bacterial loads for containment in the integrated model mimic trajectories reported from *in vivo* studies (Lin et al., 2014).

To provide a quantitative comparison between the two ABM models we compare the extracellular bacterial load of the re-implemented standard ABM and the physiological-based model for each of the three scenarios: transient containment (Figures 3A–D), containment (Figures 3E,F), and dissemination (Figures 4A,B) using six simulations in all scenarios except the transient containment scenario for the ABM-ST model, which uses five (Figures 5A–C, respective). Figure 5 shows trajectories from each of the simulation runs and the average outcomes of the runs per scenario. The average maximum values for the extracellular *Mtb* levels for the ABM-PHYS models (shown on the left vertical axis) range from approximately 2 × 10^2^ CFUs for containment to 1 × 10^3^ CFUs for transient containment and dissemination. Comparative extracellular bacterial loads for the ABM-ST model range approximately two orders of magnitude higher than their counterpart in the ABM-PHYS (shown on the right vertical axis). The extracellular *Mtb* levels continually increase for the ABM-ST model, with evidence of plateauing for the transient containment and dissemination outcomes (Figures 5A,C). Conversely the ABM-PHYS model exhibits a characteristic peak in extracellular *Mtb* levels shortly preceding 40 days post infection, followed by decreasing bacterial levels for both the transient containment and containment outcomes or increasing levels for the dissemination outcome. The ABM-PHYS model reaches steady state levels for each of the scenarios, with only the containment scenario nearing an effectively zero extracellular bacterial load. We compared the results of both models to the published plots of extracellular *Mtb* from the Segovia-Juarez et al. (2004) (Figure 9 in Segovia-Juarez et al., 2004) and Ray et al., 2009 models (Figure 3A in Ray et al., 2009). The general behavior of increasing extracellular bacteria is comparable to the trajectory of dissemination reported by Segovia-Juarez et al. (2004) and containment reported by Ray et al. (2009). However, the level of extracellular bacteria in our implementation of the standard ABM (ABM-ST) using the parameters given in Supplementary Table 1 is an order of magnitude higher than the levels depicted in the Segovia-Juarez et al. (2004) study for the dissemination scenario (approximately 4 × 10^4^ vs. 4.8 × 10^5^ at 200 days post infection) and for the bacterial levels reported by Ray et al. (2009) for containment (approximately 1 × 10^3^ vs. 3.6 × 10^4^ at 200 days post infection in our model). In comparison to the results reported in both of these studies, the characteristic trajectories and day 200 extracellular bacteria levels are notably different for the physiologically based ABM (ABM-PHYS), which is approximately 1.4 × 10^2^ for transient containment, 1.5 × 10^1^ for true containment, and 7.6 × 10^2^ for dissemination. However, the ABM-PHYS' containment trajectories have the same characteristic shape as the bacterial CFU counts presented by Lin et al., for the macaque *Mtb* infection model (see Lin et al., 2014, Figure 4). The physiologically-based ABM's containment clearly exhibits an initial build up of CFU in the granuloma until significant activation of the adaptive immune response and inflammatory mediators occurs, and then extracellular bacterial levels taper off over the remaining infection cycle.

#### Comparison of *in silico* and *in vivo* models of infection

**Figure 6:**
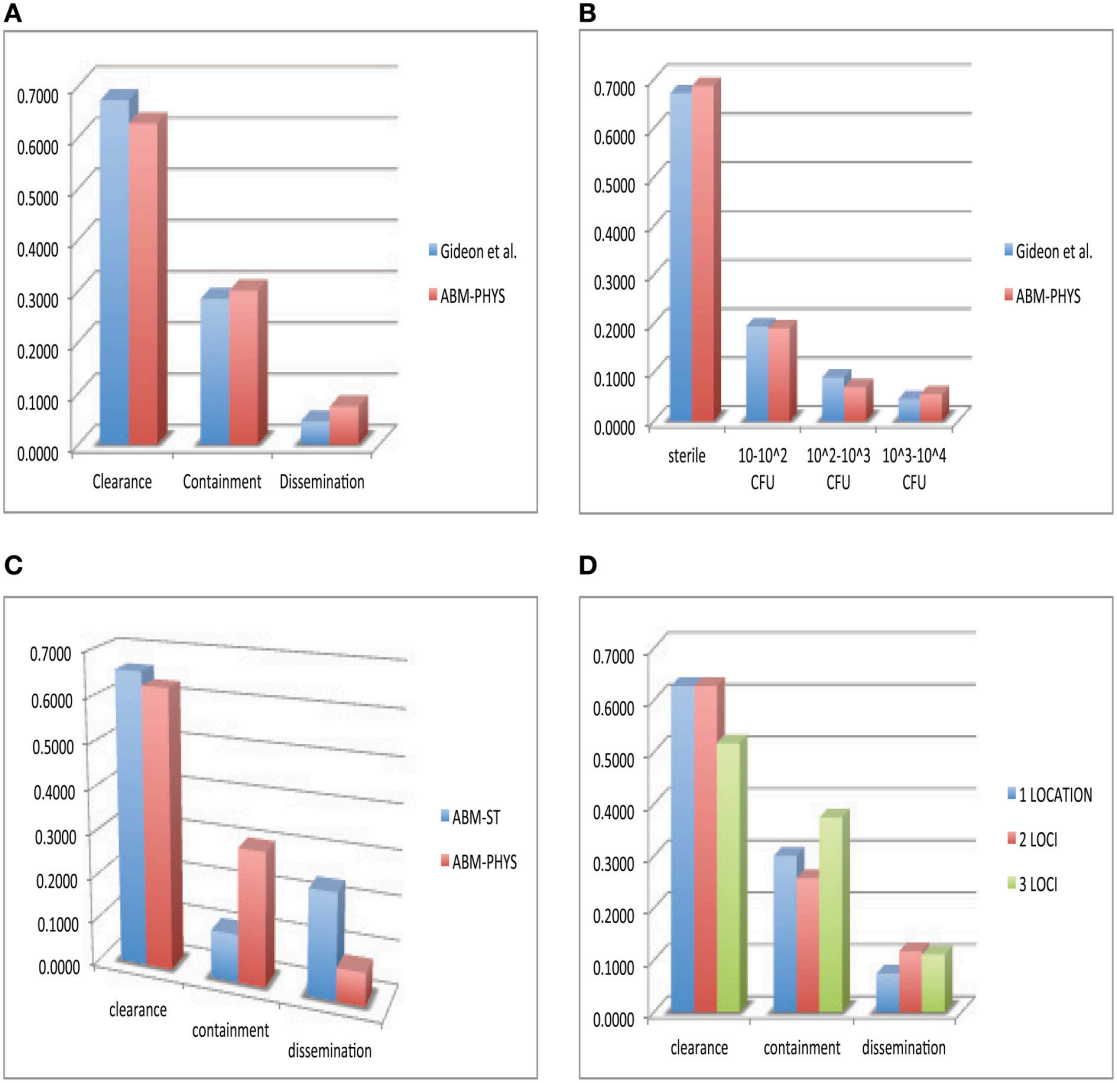
**Comparison of *in vivo* and *in silico* phenotypic outcomes**. Comparison of Gideon et al. (2015) experimental data (blue) and the ABM-PHYS model with oxygen dynamics (red): relative distribution of each qualitative outcome—clearance, containment and dissemination **(A)** distribution of lesions based on bacterial load **(B)** comparison of ABM-ST (blue) and the ABM-PHYS model (red) based on the distribution of qualitative outcomes **(C)** distribution across qualitative outcomes for 1,2, and 3 loci models of the ABM-PHYS model **(D)**. *In silico* outcomes based on multisample averages of 300 total simulations.

In the development of our model and similar to standard practice for empirical studies, we use human data when available, followed by non-human primate data, and other animal models of disease for model construction and comparative validation. Using our simulation data set consisting of 300 individual sample runs, we compare *in vivo* and *in silico* distributions of bacterial load across the three qualitative outcomes: clearance, containment and dissemination (Figure 6). Figure 6A shows the distribution of outcomes and their characteristic bacterial loads from the ABM-PHYS model (red) compared to *Mtb* infected macaques from the Gideon et al. study Gideon et al. (2015) (blue). Figure 1 in Gideon et al. shows the distribution of CFUs per lesion at a median of 222 days for both active and latent diseased macaques. Based on the per lesion CFUs reported in the study we classified the necropsied granulomas and divided them based upon bacterial load into qualitative outcomes. Using the data for individual lesions from the Gideon et al. study, we compared the *in vivo* CFU distribution to the distribution of CFUs for individual ABM-PHYS simulation outcomes at 200 days post-infection. We found that the distribution of CFUs per granuloma for the ABM-PHYS model of infection is statistically the same as the distribution of granulomas associated with latent macaques in the Gideon et al. study (*p* ≈ 0.81, see Figure 6B). When we compare the outcomes from our variable parameter study, where parameters are varied across the range of values for the healthy human, our *in silico* outcome distribution as in the latent disease case for the macaques. Figure 6A shows the distributions grouped by qualitative outcome. The distributions are not statistically significantly different (*p* ≈ 0.64).

Figure 6C shows the distribution of outcomes for ABM-ST (blue) and for the ABM-PHYS model (red) across the three qualitative outcomes. Though ABM-ST clears about 66.67 percent of *Mtb* infections, it strongly skews toward dissemination outcomes. In the ABM-PHYS model, bacterial growth rates are controlled and responsive to hypoxic conditions, so that bacterial dissemination can be modulated by host immune response and oxygen availability. An example of the impact of this phenomena is illustrated in Figures 3A,B, a transient containment scenario where ABM-ST progresses to dissemination but ABM-PHYS results in containment, at 64 days post infection. A chi-square test (*p* ≈ 1.5587E-115) confirms that the two *in silico* models produce statistically significantly different distributions, with the ABM-PHYS being closer to the actual distribution of LTBI (as determined from the Gideon et al. data). Incorporating oxygen dynamics into the system with a systems-biology model for bacterial response pushes dissemination outcomes in ABM-ST toward containment, so that the model more accurately depicts empirically observed biological mechanisms and outcomes.

Given that *Mtb* infection and disease can result in the formation of multiple granulomas Lin et al. (2014), we explored the distribution of qualitative outcomes given multiple loci of infection by varying the initial number of extracellular bacteria from 1 to 16. We modeled the infection for an infected 20 × 20 μm cellular grid. There are observable differences in response depending on the number of initial bacteria, as presented in Figure 6D. There is a significant difference between the two loci and the three loci distributions (*p* = 0.00776), in the form of much higher containment and lower clearance outcomes. The three loci distribution was also statistically significantly different (*p* = 0.0205) from the ABM-PHYS single locus model, while the two loci distribution did not differ significantly (*p* = 0.1369) from the single locus model. The number of dissemination outcomes is comparable for the two loci and three loci models.

### Host-mediated oxygen depletion and *mtb* adaptive response

We investigated the ability of the ABM-PHYS model to mechanistically link the host's physiological response and modulation of oxygen to *Mtb* metabolic fitness (Supplementary Figures 3, 4). Supplementary Figure 4 presents averages (over 300 simulations with parametric variations) for extracellular and intracellular bacterial load. We compare the four main outcomes: clearance, containment, transient containment, and dissemination. With respect to bacterial growth rates, recruitment of macrophages and oxygen depletion rates, transient containment scenarios are characteristically closer to dissemination as opposed to a true containment scenario. This can be seen in Supplementary Figures 4, 5. Figure 5, Supplementary Figure 4B show that early transient containment behaves on average more like containment with respect to the change in extracellular bacteria over time and with respect to the recruitment of macrophages. In Supplementary Figure 4 transient containment bacterial loads (a and b) are initially comparable to the containment level but approach dissemination levels after 180–200 days. The containment scenario results in significantly lower total bacteria with near zero growth rates for extracellular bacteria when compared to the transient containment and dissemination scenarios. Growth rates are shown in (Supplementary Figures 4C,D), NAD/NADH ratio (fitness measure—Supplementary Figures 4E,F) and ATP levels (Supplementary Figures 4G,H) across the four qualitative outcomes. Supplementary Figure 4I shows the average oxygen depletion rates across the grid; it can be seen that the depletion rates in our human lung model is on average comparable to the range of oxygen depletion rates observed in the Wayne NRP model with depletion rate < 0.5, which allows adaptation of *Mtb* to low oxygen conditions encountered during the first (NRP1) and second (NRP2) stages of NRP.

Supplementary Figure 5 shows the average growth rates for extracellular (a) and intracellular (b) bacteria for the center grid cell in the four qualitative scenarios, with growth rate consistently highest for dissemination followed by the transient containment scenario. In Supplementary Figure 5C we show the physiological environment correlating to the growth rates in Supplementary Figures 5A,B. It can be seen that containment is on average hypoxic (<2% oxygen), while the other scenarios have notably higher levels of oxygen with clearance and dissemination having the highest levels of oxygen. These results show that, with respect to the bioavailability of oxygen, the physiological microenvironment of containment granulomas within the human lung are largely hypoxic when compared to disseminated or cleared infections (Supplementary Figure 5C).

*In vitro* studies of *Mtb* persistence suggest that depletion dynamics vs. bioavailability of oxygen is a key determinant of *Mtb* persistence (Wayne and Hayes, 1996). Using simulation data from our three scenarios, oxygen depletion is calculated as the change in percent of oxygen utilization over change in time and evaluated across the entire grid, Supplementary Figure 4I, and at the center of the granuloma, Supplementary Figures 5C,D. Across the grid average oxygen depletion rates are higher for the containment model, with the transient containment showing higher depletion rates than the dissemination scenario only between approximately 50 and 100 days post infection. The failure to maintain sustained oxygen depletion contributes to the deterioration of the transient containment granuloma into a dissemination outcome at time points greater than 200 days post infection. Oxygen depletion rates at the center of the granuloma range from 0 to 0.5 for most cells in the ABM-PHYS model, Supplementary Figure 5D. The range of depletion rates seen in the model falls within the range of depletion rates observed in the slow-stirred condition in the *in vitro* Wayne NRP model (Wayne and Hayes, 1996). The containment granuloma features enduring hypoxic regions, Supplementary Figure 5C, and eventually these regions reach and surpass reduced oxygen levels associated with NRP stage 1. Dissemination and transient containment have a greater propensity to show transient hypoxic periods, Supplementary Figure 5D. Clearance scenarios show minimal oxygen depletion, which we attribute the clearance of *Mtb* to a non-oxygen associated, robust immune response-mediated killing of bacteria, Supplementary Figure 5D.

Failure to maintain a sustained hypoxic environment may enable *Mtb* to proliferate in the extracellular and intracellular environment during dissemination, Supplementary Figures 5A,B. However, sustained oxygen depletion rates during containment result in reduced extracellular and intracellular bacterial growth rates, but the oxygen depletion rate still gives the bacteria time to metabolically adjust to changes in oxygen levels in its local environment. Similar to the Wayne NRP slow-stirred model, bacteria are not killed off as in the vigorously shaken/rapid oxygen depletion scenario, but rather they persist within the granuloma. Persistent bacteria can maintain their relative metabolic fitness. Intracellular NAD/NADH ratios of bacteria during containment start off initially lower than the dissemination outcome for simulation time < 100 days and reach levels comparable to disseminating bacteria post 100 days, Supplementary Figures 4E,F. Furthermore, the intracellular ATP levels of bacteria during containment are higher when compared to the dissemination bacteria, with the *Mtb* in containment outcomes having notably higher levels of ATP than *Mtb* in dissemination outcomes, Supplementary Figures 4G,H. While intracellular containment bacteria are potentially more metabolically fit than the intracellular disseminating bacteria, the extracellular containment bacteria exhibit a reduced fitness profile. The NAD/NADH and ATP levels are lower in containment bacteria than in disseminating bacteria post 60 and 80 days simulation time, respective. The transient containment bacteria have a markedly different metabolic response. In general extracellular and intracellular transient containment bacteria have the lowest ATP levels, however the NAD/NADH levels for both extra- and intracellular containment bacteria are distinctively higher than both containment and dissemination bacteria post 100 days. During clearance there are very few remaining bacteria on the grid, Supplementary Figures 4E,F inset, potentially resulting in an inflated average NAD/NADH ratio. However, the remaining bacteria during the clearance scenario have very low comparable levels of ATP, Supplementary Figures 4G,H, reducing their likelihood of persistence or proliferation.

### Influence of oxygen depletion on the course of tuberculosis disease

**Figure 7:**
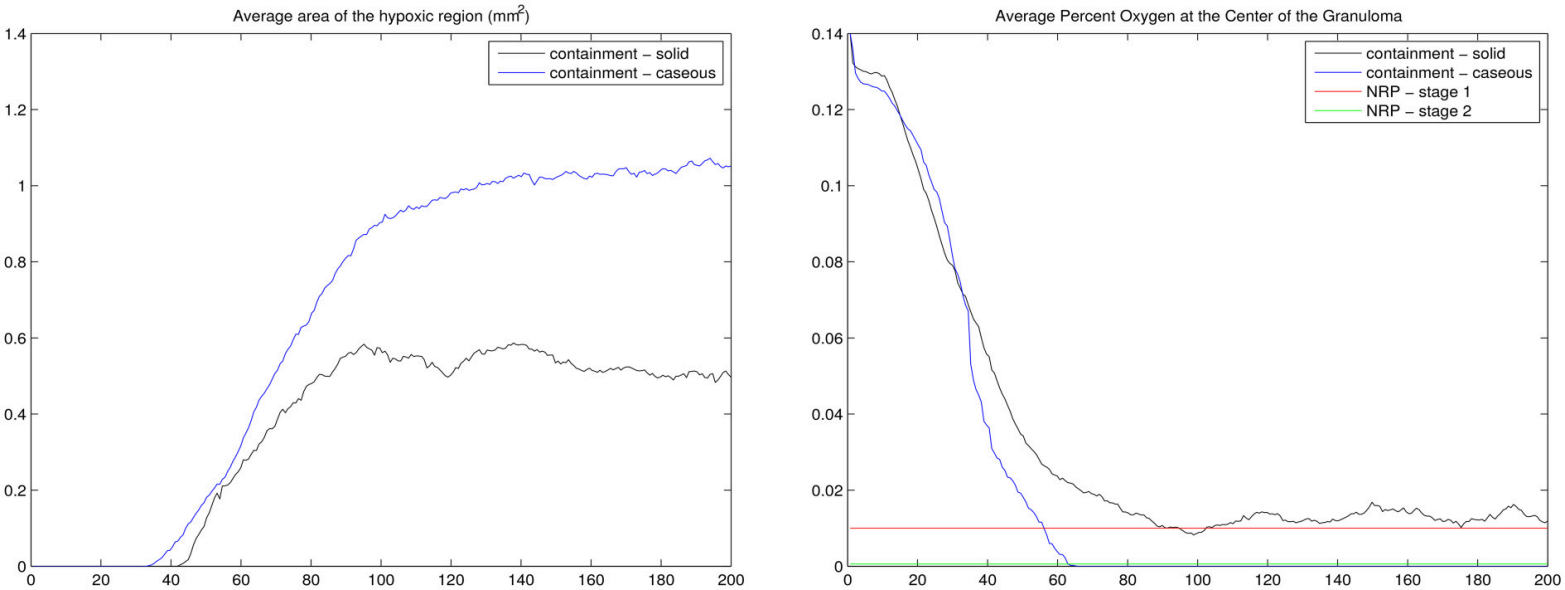
**For the containment granuloma: average area of the hypoxic region in *mm*^2^ (Left) and the average percent of oxygen in tissue at the center of the granuloma (Right)**. Containment granulomas were divided into two groups: caseous and solid. NRP stages (Wayne and Hayes, 1996) are notated (right). Results based on 20 simulation runs.

The results of our ABM-PHYS model support the hypothesis that the hypoxic environment of the human lung contributes to the shiftdown of *Mtb* toward persistence during the formation of a containment granuloma. Figure 7 (left) shows the average size of the hypoxic region for the containment granuloma. Simulated granulomas were separated into two groups: solid and caseous in order to compare outcomes and oxygen-related characteristics of granulomas in our *in silico* model to *in vivo* granulomas described by Via et al. (2008). If a simulated granuloma contained caseous cells, it was classified as caseous and in the absence of these, as solid. On day 56 post-infection, which correlates to when tissue samples from *Mtb* infected animals were collected in the *in vivo* Via et al. study, in the simulated ABM-PHYS model the average size of the hypoxic regions are on the order of 0.2490 and 0.2135 mm^2^ for caseous granulomas and solid granulomas, respectively. The size is in close agreement with the *in vivo* experimental study which reported a hypoxic region on the order of 0.36 mm^2^ in rabbit lungs. Via et al. note that all caseous granulomas were positive for pimonidazole hydrochloride (PIMO) staining, a hypoxia indicator, while only 32% of the solid granulomas were positive for PIMO activation.

**Figure 8:**
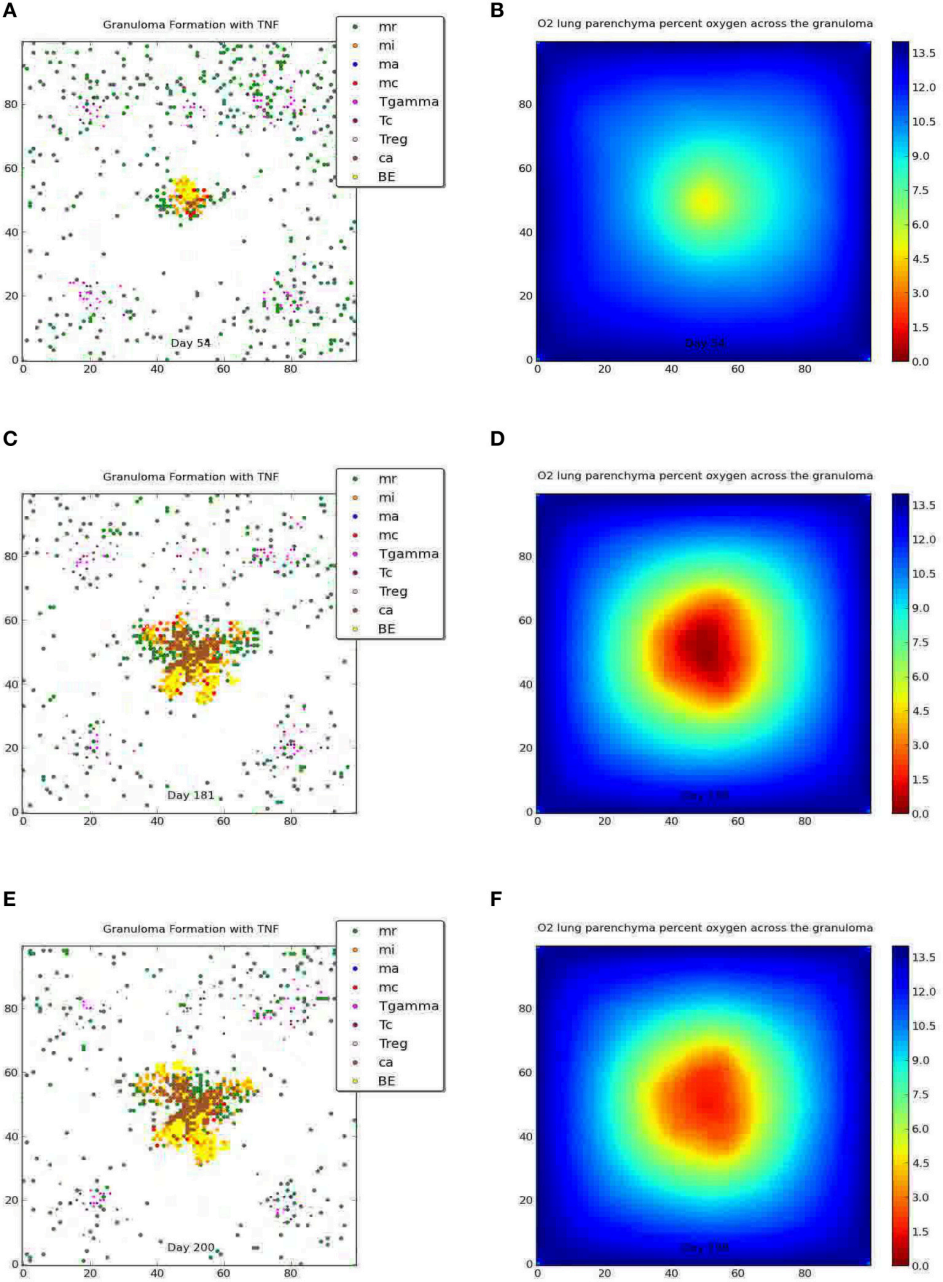
**Dissemination and the corresponding oxygen field at 54, 181, and 200 days post-infection**. Severe hypoxia is seen on day 181, which resolves by day 200.

The Wayne NRP studies suggest that microenvironments that gradually become hypoxic enable *Mtb* to transition from an NRP1 stage to an NRP2 stage, resulting in the establishment of non-replicating, persistent *Mtb* (Wayne and Hayes, 1996). To determine if and to what extent the physiological immune response results in a microenvironment similar to the environment observed in the Wayne *in vitro* studies, we analyzed the average percent oxygen in tissue for the two categories of containment granuloma (Figure 7, right). It is seen that the oxygen levels in the simulated caseous containment granulomas are on average comparable and transition similar to the Wayne model's NRP stage 1 and NRP stage 2. Solid granulomas average slightly higher than 1% oxygen. Thus *Mtb* transitioning into a persistent state is more likely in the case of caseous containment granulomas. In comparison we show in Figure 8 a tableaux of transient hypoxic states for a dissemination outcome over time. Severe hypoxia is seen on day 181, but resolves by day 200 with concomitant bacterial growth, demonstrating that sustained hypoxia is needed for bacterial containment.

**Figure 9:**
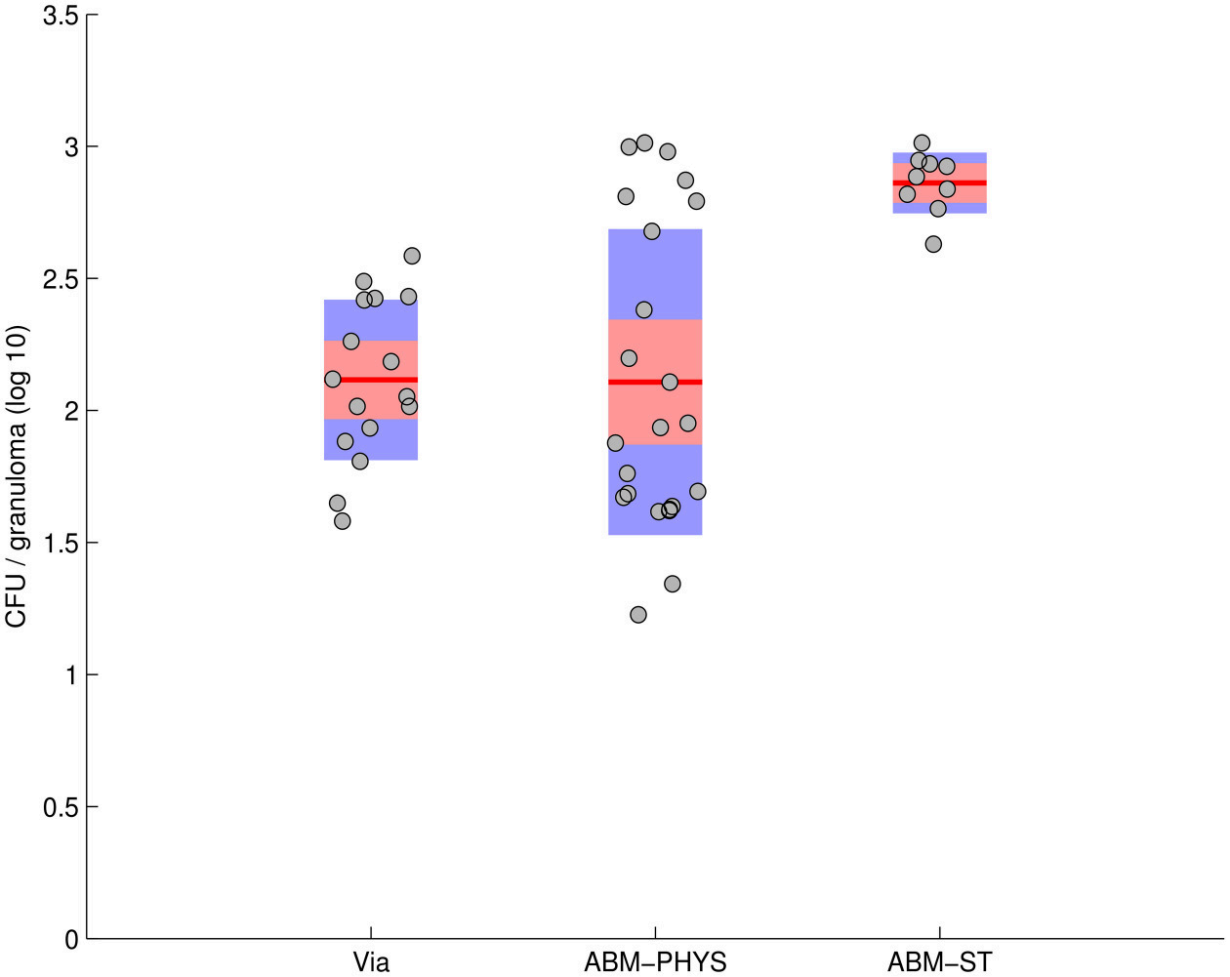
**Total CFU for *M*. *bovis* untreated infected rabbits on day 63 (Via et al., 2008—bar 1) compared with total CFU resulting from the *in silico* model of *Mtb* infection produced by the ABM-PHYS and the ABM-ST model using the same specific growth rate of *Mtb* for both models**. *In silico* results are based on 10 simulations for ABM-ST and 22 simulations for ABM-PHYS.

Statistical comparison of the *in silico* ABM-PHYS, *in silico* ABM-ST, and *in vivo Mtb* infection models indicates that our ABM-PHYS model provides a more comparable reproduction of bacterial load over time than the ABM-ST model. Using published experimental data from the *in vivo* study (Via et al., 2008) of 16 NZW rabbits infected with *M*. *bovis*, we compare the number of mycobacterial CFUs observed in *in vivo* granulomas to the number of CFUs in our simulated granuloma. The data extracted from Via et al. show the bacterial CFU per individual 1- to 1.5-mm granuloma at 63 days post infection (5 weeks of housing and 28 subsequent days of placebo treatment) for the experimental control group, see the first bar in Figure 9. In the same figure we comparatively plot the distribution of total bacteria at day 63 for 23 simulated containment granulomas produced using the ABM-PHYS model with variable specific growth rate (μ-max = 0.006649 per hour using the default in Table 1; see bar 2 in Figure 9) and 10 simulated containment granulomas generated using the ABM-ST model (Figure 9, bar 3), with the same parameters but using fixed bacterial growth rate and no oxygen dynamics. It can be seen that the ABM-PHYS model's estimated mean CFU per granuloma and the associated variance more closely matches the *in vivo* data than the ABM-ST model. Using the Kolmogorov–Smirnov (K–S) test, which is a non-parametric test to determine whether two distributions are statistically significantly different, we found that the ABM-PHYS model's total bacterial distribution is not statistically significantly different from the experimental control distribution in Via et. al. (*p* = 0.2702). For the ABM-ST the simulated distribution is statistically significantly different from the experimental data (*p* = 3.8254e-06). The ABM-PHYS vs. ABM-ST results from the K–S test were also statistically significantly different (*p* = 0.0017) from one another. The results of the K–S test suggests that the ABM-PHYS model more accurately captures the mean and variance of the experimental control data for the containment scenario in the Via et. al. study. Though the ABM-PHYS features greater variance in total bacterial load, it produces a more physiological and immunologically accurate correlation of the impact of granuloma formation on the macroscopic host environment and on the microscopic extracellular/intracellular bacterial load. Allowing growth rates of *Mtb* to fluctuate in response to local environmental oxygen conditions appears to more accurately portray actual infection dynamics.

### Uncertainty and sensitivity analysis—UA, SA

We used the freely available software DAKOTA (Adams et al., 2010) developed by Sandia National Laboratories to perform both uncertainty and sensitivity analysis for our ABM-PHYS model (see Table 5). In Supplementary Figure 6 we show the partial regression correlation coefficients (PRCC) over 200 days post-infection for the statistically significant multiscale model driver variables, with the measured outcome variable being the level of extracellular bacteria. An increased level of extracellular bacteria suggests a failure in immune response and results in *Mtb* dissemination. Therefore, extracellular bacterial load serves as a valid quantitative metric that correlates to active TB disease (see Supplementary Figures 4A,B for a comparison of total bacterial load to extracellular bacterial load). To identify statistically significant input variables for our ABM-PHYS model we performed a variable parameter study. In order to reduce aleatory uncertainty the model was run for three iterations using a different random number seeds at the beginning of each iteration and we ran the three-iteration cycle 100 times resulting in *N* = 300 simulation runs. Each set of three simulation outcomes were averaged and the PRCC produced (see Marino et al., 2008 for further discussion). This gave us a total of 100 sample outcomes (generated from 300 individual simulations), each an average of three simulations ran for each of the 100 input parameter sets varied over the ranges described in Table 2 (Ray et al., 2009). We used Dakota to generate the resulting PRCC. Supplementary Figure 6A shows the significant variables with clearance outcomes included (*p* < 0.05) using our original *N* = 300 simulation runs and Table 6 lists the statistically significant model parameters with all outcomes considered, together with *p*-values to indicate level of significance. We found that the chemokine diffusion coefficient and CCL5 threshold impacts infection dynamics very slightly (negatively and positively correlated, respective) when the clearance scenarios are included. We observed that pulmonary blood sources and residual lung volume are positively correlated with extracellular bacteria, suggesting that an increase in pulmonary blood sources in the lung may provide increased oxygen to the parenchyma, thus enabling growth and development of extracellular bacteria. Increased residual lung volume provides more oxygen to tissues resulting in a more aerobic environment, which is more amenable for extracellular bacterial growth. Macrophage and bacterial O2 consumption parameters are significant drivers in our model, both negatively correlated with extracellular bacteria. Higher oxygen consumption by macrophages and high consumption by a single bacterium leaves less O2 available for other bacteria. The faster oxygen diffuses through tissues, which is determined by the O2 diffusion parameter, the more oxygen is readily available to extracellular bacteria, hence the positive correlation with bacterial load. Since naturally occurring oxygen is comprised of three stable isotopes, O2 can persists longer than chemokines/cytokines in the system. A high probability for T cell movement into a macrophage containing compartment facilitates the activation of macrophages and enables the formation of a more tightly controlled granuloma. Therefore, a high Tmove is negatively correlated with bacterial load and leads to lower extracellular bacteria. Finally high growth rate (μ-max) of *Mtb* has a positive impact on extracellular bacteria levels.

**Table 5:** **Containment and dissemination parameters for simulation runs used in sensitivity analysis**.

**Description**	**Units**	**Containment (Mean)**	**Containment (Min)**	**Containment (Max)**	**Dissemination (Mean)**	**Dissemination (Min)**	**Dissemination (Max)**
Probability a resting macrophage kills a bacterium in his compartment	Per 10 minutes	0.0541	0.0131	0.0972	0.0486	0.0162	0.0781
Probability a macrophage is recruited from a vascular source	Per 10 minutes	0.0661	0.0213	0.1405	0.0631	0.0188	0.1294
Probability a T cell is recruited at a vascular source	Per 10 minutes	0.0542	0.011	0.137	0.109	0.0754	0.1415
Proportion of T cells recruited that are regulator T cells	Per 10 minutes	0.0763	0.0125	0.1562	0.152	0.0208	0.196
Chemokine diffusion constant	cm^2^/per second	6.00E-08	1.86E-08	1.07E-07	7.89E-08	3.29E-08	1.05E-07
Chemokine halflife	Hours	1.45	0.64	2.24	1.49	0.77	2.27
CCL5 secretion	No. molecules secreted hourly	2.78E+05	7.53E+04	4.59E+05	2.60E+05	6.34E+04	4.37E+05
TNF diffusion constant	cm^2^/per second	4.31E-08	1.99E-08	1.01E-07	7.87E-08	2.56E-08	1.12E-07
TNF halflife	Hours	7.12	1.19	11.17	5.22	2.31	8.42
Probability a macrophage undergoes apoptosis	Per 10 minutes	0.13	0.08	0.19	0.1	0.04	0.18
Threshold required for macrophage recruitment	Checked every ten minutes	6.96E+05	1.97E+05	1.44E+06	1.08E+06	8.27E+05	1.45E+06
Oxygen in lung tissue due to pulmonary blood volume	Steady-state number of molecules	5.68E+08	2.81E+08	9.42E+08	5.76E+08	3.43E+08	8.39E+08
Oxygen in lung tissue due to residual volume in lung	Steady-state number of molecules	9.53E+08	4.02E+08	1.35E+09	8.61E+08	6.09E+08	1.09E+09
Oxygen consumption by a resting macrophage	Number of molecules per 1 breath	7.19E+07	6.16E+07	8.15E+07	7.55E+07	5.88E+07	9.46E+07
Oxygen consumption by bacteria	Number of molecules per 1 breath	8.77E+05	5.50E+05	1.15E+06	8.07E+05	4.90E+05	9.98E+05
Oxygen diffusion coefficient	cm^2^/per second	5.83E-05	5.98E-06	1.10E-04	5.03E-05	5.38E-06	8.82E-05
TNF threshold necessary to activate a macrophage	Checked every 10 min	1.82E+05	6.68E+03	3.18E+05	2.02E+05	4.09E+04	3.44E+05
Probability an infected macrophage becomes activated	Per 10 minutes	2.92E-02	3.37E-04	7.52E-02	3.05E-03	3.14E-04	8.91E-03
Probability a T cell moves into a compartment occupied by a macrophage	Per 10 minutes	1.65E-03	1.90E-05	8.18E-03	2.91E-02	1.03E-05	8.88E-02
TNF/chemokine threshold for T cell recruitment at a vascular source	Checked every 10 min	1.73E+04	1.17E+03	8.13E+04	7.73E+03	2.64E+03	1.55E+04
TNF/chemokine threshold for macrophage recruitment at a vascular source	Checked every 10 min	1.32E+04	1.24E+03	8.76E+04	7.19E+03	1.39E+03	2.30E+04
Lower threshold for recruitment of CCL5	Checked every 10 min	3.09E+05	1.75E+04	7.18E+05	1.28E+05	1.69E+04	4.40E+05
Upper threshold for recruitment of CCL5	Checked every 10 min	1.83E+05	1.22E+04	6.79E+05	3.68E+04	1.58E+04	6.60E+04
TNF secretion	No. molecules per 10 min	5.33E+06	9.50E+04	2.87E+07	3.82E+06	2.09E+04	1.67E+07
Effect of TNF on resting macrophage recruitment	Checked every 10 min	225.53	12	706.96	255	16.39	875.23
Maximum specific growth rate	Hourly	0.0127	0.0024	0.0386	0.0343	0.0305	0.0379
Maximum specific death rate	Hourly	0.0004	0.0001	0.0008	0.0006	0.0004	0.0007

**Table 6:** **Significant Partial Rank Correlation Coefficients for the integrated multiscale model of oxygen-modulated host response to *Mtb* infection**.

**Variable**	**Max/Min **p**-value**	**Explanation of significance**
Chemokine diffusion constant	–	Fast diffusion of chemokine can lead to an increased rate of signaling to neighboring cells, positively
		affecting macrophage recruitment toward the site of infection and leading to lower levels
		of extracellular bacteria (EB).
Pulmonary blood source	+	More pulmonary blood provides increased oxygen to the lung thus higher EB level can persist.
Residual volume	+	Higher residual volume provides more oxygen to tissues, thus is a more friendly environment for EB.
Macrophage O2 consumption	–	Higher oxygen consumption by macrophages leaves less oxygen available for bacteria,
		thus is negatively correlated to EB.
O2 bacterial consumption	–	High consumption by a single bacterium leaves less O2 available for other bacteria.
Oxygen diffusion coefficient	+	The faster oxygen diffuses through tissues, the more oxygen is readily available to EB.
Tmove	–	Enables a more tightly controlled granuloma facilitating activation of macrophages, therefore lower EB.
CCL5uthreshold	+	Higher threshold implies less recruiting of macrophages at vascular source sites, so more EB may persist.
mu max	+	Higher growth rate of EB has positive impact on EB levels.

Positive correlations with extracellular bacteria level:(+) = p ≤ 0.025;(++) = p ≤ 0.01;(+++) = p ≤ 0.001;Negative correlations with EBL:(−) = p ≤ 0.025;(–) = p ≤ 0.01.

**Table 7:** **Explanation of simulation data sets used to generate figures of aggregate results and comparative outcome for ABM-PHYS and ABM-ST**.

**Parameter type**	**Analysis type**	**Number of simulations**	**Figure numbers**
Variable parameter	Comparison of average phenotypes across	*N* = 300	Supplementary Figures 3–5
	all outcome categories (clearance, containment, dissemination).		
	Comparison of statistical distribution of outcomes	*N* = 300	Figure 6
	for *in vivo*, ABM-PHYS, and ABM-ST.		
	Identification of statistically significant model	*N* = 300	Supplementary Figure 6A
	parameters across all outcomes.		
	Identification of statistically significant model	*N* = 41 (of 150)	Supplementary Figure 6B
	parameters for containment and dissemination outcomes.		
Fixed parameter	Outcome specific comparison of bacterial load phenotype.	*N* = 5 to 6	Figure 5
^*^ Data sets where some	Comparative analysis of the hypoxic region of	*N* = 20(^*^4)	Figure 7
*are from variable*	containment granulomas.		
*parameter runs*	Comparison of the bacterial load in *in vivo*	*N* = 10,22(^*^1)	Figure 9
	vs. *in silico* containment granulomas.		
	Comparison of *Mtb* gene expression for containment	*N* = 20 (^*^10)	Supplementary Figure 2
	and dissemination outcomes.		

Aggregate or average results are generated based on the N = 300 LHS simulation run set or from fixed parameter simulation sets (parameters used for fixed parameter studies are listed in Appendix 4 in Supplementary Material). Figure 6, Supplementary Figures 3–5, 6A are generated using the N = 300 simulation set for both the standard and integrated ABM models, where we categorized outcomes as described in Appendix 1 in Supplementary Material. Supplementary Figure 6B is produced using 41 of 150 simulation results (over the same parameter space as the N = 300 simulation set) in order to investigate which significant parameters result when we exclude the clearance outcomes. Figure 5, which compares the number of extracellular bacteria in the standard ABM to the integrated ABM, is generated from 5 to 6 fixed parameter simulations for each scenario. In the remaining figures we used fixed parameter simulation runs to generate a sufficient number of containment samples for comparative analysis and characterization of the containment response: Figure 7 results are based on 20 simulations representing 6 solid containment granulomas and 14 caseous containment granulomas (of the 20 simulations, 4 were from the N = 300 sample run set and 16 from fixed parameter simulation runs); Figure 9 results are based on 10 fixed parameter simulations for the standard ABM and 22 simulations for the integrated multiscale ABM (of the 22 simulations, 1 was from the N = 300 sample run and 21 from fixed parameter simulation runs); Supplementary Figure 2 results are based on 20 simulations (10 were from the N = 300 sample run set and 10 from fixed parameter simulation runs).

Given the large number of clearance outcomes represented by the *N* = 300 (100 average outcomes) simulation run, we generated a non-averaged simulation set to determine which parameters are statistically significant if we consider only the non-clearance outcomes. To determine the PRCCs for the containment and dissemination outcomes, we ran 150 individual simulations of the model over our input parameter space using different random number seeds. The 150 runs resulted in 41 non-clearance, non-averaged outcomes, which ensured that we retained a reasonable number of degrees of freedom after removing the clearance outcomes to determine statistical significance. We used the rules in Appendix 1.1 in Supplementary Material to categorize the outcome as clearance vs. non-clearance. (See Tables 1, 2 for the ranges used in the Latin-hypercube sampling (LHS) analysis for each of the variables of interest.) We performed our PRCC analysis using the method described by Marino et al. (2008) and associated PRCC calculation tools freely available via download (Marino et al., 2008). Supplementary Figure 6B shows the significant variables without the clearance outcomes included in the analysis (*p* = 0.05) using *N* = 41 of 150 set of individual simulation runs. The significant inputs include: probability that a macrophage kills bacteria, which is negatively correlated to extracellular bacterial (ECB) load; probability of macrophage recruitment, which is positively correlated with ECB levels possibly due to crowding effects within the granuloma and the inability of T cells to migrate to the center and activate macrophages, as described well in Segovia-Juarez et al. (2004). Increased T reg cell recruitment to ECB-containing regions may result in a lower probability of cytotoxic (killer) T cell recruitment, thus the positive correlation of the T reg recruitment with ECB. TNF diffusion constant and TNF half-life are positively correlated with ECB, at later stages of the infection, which is plausible given TNF's roles in inducing apoptosis of infected macrophages (Wajant et al., 2003). Combined TNF/chemokine threshold for T cell recruitment (r.T) is negatively correlated with extracellular bacterial load as a low threshold leads to increased T cell recruitment and consequentially an increase in the number of activated macrophages able to eliminate the pathogen. The final significant parameter, TNF secretion, is negatively correlated with ECB early on in the infection, which would be expected given TNF's pro-inflammatory role (see Ray et al., 2009). However, it is slightly positively correlated with ECB around 100 days post infection, which may be attributed to chronically infected macrophages and T cell populations continual production of TNF during the dissemination scenario.

## Discussion

Using our ABM-PHYS model of TB disease, we explored the correlation between host immune response, physiological response with respect to oxygen, and outcome of infection. While the model architecture determined the general interaction between model components, we used uncertainty quantification methods to explore the parameter space and discover emergent system properties that correspond to bacterial clearance, containment/latency, or dissemination.

Using outcomes from our simulation model our analysis has shown significant correlations between the oxygen input variables and the extracellular bacteria levels (ECB) when all outcomes are considered (Table 6; Supplementary Figure 6A). Notably parameters such as chemokine diffusion constant, pulmonary blood source, residual volume, macrophage O2 consumption, O2 bacterial consumption, oxygen diffusion coefficient, Tmove, CCL5uthreshold, and μ_*max*_ (Table 6) were significantly correlated with the amount of extracellular bacteria, which was used to classify simulation outcomes. The majority of these parameters are related to physiology-dependent oxygen availability or immune-dependent modulation of oxygen physiology. Therefore, the model demonstrates that oxygen-related physiological characteristics (pulmonary blood source, residual volume, oxygen diffusion coefficient, baseline macrophage O2 consumption) combined with immune-related physiological characteristics that bring oxygen-consuming cells to the site of infection (chemokine diffusion constant, Tmove, CCL5 threshold) integrate to determine how quickly hypoxic regions occur and how long they are maintained. The rate of hypoxic onset and duration in turn impacts *Mtb* oxygen relevant characteristics (bacterial O2 consumption, bacterial growth rate/μ_*max*_, and persistence). The observed connection between physiology, immune response, oxygen gradients and infection outcome, demonstrates a structural host response mediated, oxygen-dependent immunological contribution to *Mtb* infection outcome. However, when we only consider the containment, transient containment and dissemination scenarios (Supplementary Figure 6B), seven significant inputs appear, none directly related to oxygen. The significant parameters for the non-clearance outcomes suggest a more macrophage/TNF centric response to infection, with reduced pro-inflammatory response when compared to clearance outcomes. The absence of O2 drivers in the non-clearance outcomes provides further evidence supporting the importance of the oxygen dependent physiological immune response in eliminating *M. tuberculosis*.

### The transient LTBI response

Another unique aspect of this work is that, to our knowledge, our model represents the first integration of *Mtb* metabolic dynamics into the ABM modeling framework to enable the exploration of bacterial response to host dynamics and physiological oxygen dynamics concurrently. The integration and the number of bacterial cells we model necessitated the use of an integrated software platform to serially execute the cellular model, while we ran the intracellular TB model using BioXyce. The integration of the host–pathogen interaction and physiological responses enabled the exploration of both molecular and cellular mechanisms that contribute to granuloma formation in tuberculosis.

We used the derivatives of the external bacterial load and recruitment of new macrophages for our mathematical classification of the four qualitative outcomes, including transient containment (see Supplementary Figures 3–5, Appendix 1 in Supplementary Material). Previous models of TB focus on the three recognized clinical outcomes of infection: disease clearance, granuloma formation and containment, or pathogen dissemination. Our discovery of the transient containment category of outcomes is a new insight from our work that has not been addressed in prior theoretical models of TB infection and granuloma formation, and is an observation that can contribute to our understanding of *Mtb* reactivation. Reactivation of *Mtb* from a granuloma has been investigated in other models by various means such as simulating the addition of an exogenous chemical agent that interferes with immune response. However, we identified and computationally observed transient containment events as an emergent property where granulomas seem to fail without exogenously introduced factors and the presumably contained bacteria moves toward reemergence and dissemination. Our observation and characterization of this class of outcomes can point to immunological differences that contribute to chronic disease and of great importance, factors that may lead to the breakdown of granulomas and potentially disease re-activation in LTBI individuals with reduced pulmonary capacity such as those with emphysema or COPD. Also the framework can be used to investigate how modulating non-host factors such as environmental oxygen changes the outcome of infection and granuloma formation. Given that relocation of TB patients to higher altitudes was previously considered therapeutic, as an example, we lowered the atmospheric oxygen constant to represent 18% oxygen (higher altitude, Supplementary Figure 7B) instead of 21% representing normal oxygen levels at sea level (Supplementary Figure 7A). The change in environmental oxygen leads to a slightly more tightly-formed granuloma with less caseous cells under 18% oxygen than the 21% oxygen levels at 200 days post-infection.

In summary, the multiscale modeling approach enables portrayal of granuloma structure, dynamics, and demonstrates the link between granuloma physiology and immunological functionality. Incorporating oxygen dynamics into the framework of granuloma simulation and integrating a systems biology model of *Mtb* allows us to capture *Mtb* biochemical response to oxygen dynamics in the bacterias immediate microenvironment, with the pathogen showing an adaptation response similar to that observed in the Wayne model during the two stages of *in vitro* NRP.

While the bacterial response is similar (in terms of persistence under varying conditions) to the Wayne model, the physiological immune response that led to the various microenvironments and simulation model outcomes were not designed into the model. The changes in the microenvironment (oxygen dynamics, dynamic onset of hypoxia, etc.) are emergent properties that were observed after categorizing the simulation results into the four outcomes (containment, clearance, etc.). The observation (Figure 7) that the oxygen dynamics for the containment outcome was similar to the Wayne NRP1 and NRP2 microenvironment is again an emergent outcome and not forced by the model. As such, these results demonstrate the importance of accounting for the physiological aspects of immune response in theoretical models of *Mtb* infection.

Our simulation model also replicates the oxygen-dependent immunological outcomes of infection observed *in vivo*, with the average size of the hypoxic regions calculated for our simulated containment granuloma correlating to that found in *in vivo* models of *Mtb* infection (Via et al., 2008). As a result our integrated multiscale model is able to more accurately capture the physiological, cellular, and molecular host–pathogen mechanisms that are key to successful host clearance of *Mtb*, host failure and *Mtb* dissemination, or bacterial persistence and onset of LTBI, a current challenge in the treatment of tuberculosis.

We have demonstrated through simulation that the structural immune response coupled with the physiological impact of oxygen is a mediating influence on the outcome of *Mtb* infection. Specifically our results have shown that including oxygen dynamics in the model enables a closer portrayal of the progression of the infection, which more accurately parallels statistical infection outcomes observed in animal models of TB disease and in WHO human LTBI rates in the population. The methods used for incorporating oxygen into a multiscale infection model is extensible to other disease models, therefore the modeling methodology we developed is not only TB specific but broadly relevant as our methods can be applied more widely to understand other host-pathogen systems.

## Author contributions

Conceived and designed the experiments: CS, EM. Performed the experiments: CS. Analyzed the data: CS, EM. Developed modeling and analysis tools: CS, SP, EM. Wrote the manuscript: CS, SP, EEM.

### Conflict of interest statement

The authors declare that the research was conducted in the absence of any commercial or financial relationships that could be construed as a potential conflict of interest.

## Supplementary material

Supplementary Figure 1 **Regression model for two genes etfA and fum: actual (blue) vs. predicted (red)**.

Supplementary Figure 2 **Gene expression for cell at the center of the grid for the containment and dissemination outcome scenarios over 200 days post-infection. ATPH gene (Left); ACEAB gene (Right)**. Inset: Voskuil data for aerobic (red) and hypoxic (green) conditions. Results represent averages over 10 simulations per outcome (5 from *N* = 300 simulation run set and 5 from fixed parameter simulation runs).

Supplementary Figure 3 **Substrates levels from the *Mtb* metabolic model 200 days post-infection**. Glycine-a; Malate-b; Isocitrate-c; Glyoxylate-d. Results represent sample averages from 300 simulation run set (number of simulations per outcome: 13 clearance, 6 containment, 5 transient containment, and 7 dissemination).

Supplementary Figure 4 **Average response of cells across the simulation grid during clearance, containment, and dissemination. (A)** Average growth rate of extracellular bacteria (inset - equilibrium containment averages); **(B)** average growth rate of intracellular bacteria; **(C)** average hourly extracellular growth rate; **(D)** average hourly intracellular bacterial growth rate; **(E)** average scaled NAD/NADH ratio of extracellular bacteria; **(F)** average scaled NAD/NADH Ratio of intracellular bacteria; **(G)** average extracellular ATP; **(H)** average intracellular ATP; **(I)** average rate of oxygen depletion across the granuloma. Averages over 300 total simulations.

Supplementary Figure 5 **Response of cell at the center of the grid for the clearance, containment, and dissemination outcome scenarios: (A) average growth rate of extracellular bacteria; (B) average growth rate of intracellular Bacteria; (C) average oxygen tension (mmHg); (D) average depletion rate**. Averages over 300 total simulations.

Supplementary Figure 6 **Significant Partial Rank Correlation Coefficients for the integrated multiscale model show the impact of oxygen dynamics and physiological host response on *Mtb* infection outcome**. Extracellular Bacterial levels are used as the outcome measure. Analysis results are based on multi-sample averages of the 300 simulation outcomes **(A)** and 41 individual non-clearance outcomes **(B)**.

Supplementary Figure 7 **Containment granuloma at 21 percent oxygen (left, A) and 18 percent oxygen (right, B)**.

Supplementary Table 1 **Parameters of the containment and dissemination run both with the ABM as standalone model and the multi scale model**.

## Abbreviations

Mtb: *Mycobacterium tuberculosis*
NRP: non-replicating persistence
TB: tuberculosis
WHO: World Health Organization
LTBI: latent tuberculosis infection
NO: nitric oxide
TNF-α: tumor necrosis factor alpha
CCL2/CCL5: chemokine (C-C motif) ligand 2/5
CXCL9/10/11: chemokine (C-X-C motif) ligand 9/10/11
ODE: ordinary differential equation
ATP: adenosine triphosphate
ABM: agent-based model
TCA: tricarboxylic acid
NAD/NADH: nicotinamide adenine dinucleotide/hydride
LHS: Latin hypercube sampling
PCC: partial correlation coefficients
PRCC: partial regression correlation coefficients
K-S test: Kolmogorov-Smirnov test
CFL: condition Courant-Friedrichs-Lewy condition
CFU: colony-forming unit
ETC: electron transport chain
IFN-γ: Interferon-γ
HIV: human immunodeficiency virus
COPD: chronic obstructive pulmonary disease
LAPACK: linear algebra package
pK: probability a macrophage kills *Mtb*
prob-recruit-T: probability a T cell is recruited at a vascular source
tao-TNF: macrophage TNF detection threshold
3D: three-dimensional.
