# Supplementary figures and images for: Oxygen Modulates the Effectiveness of Granuloma Mediated Host Response to Mycobacterium tuberculosis: A Multiscale Computational Biology Approach
Source: Front Cell Infect Microbiol. 2016 Feb 15;6:6. doi: 10.3389/fcimb.2016.00006 (PMC4753379; doi:10.3389/fcimb.2016.00006)

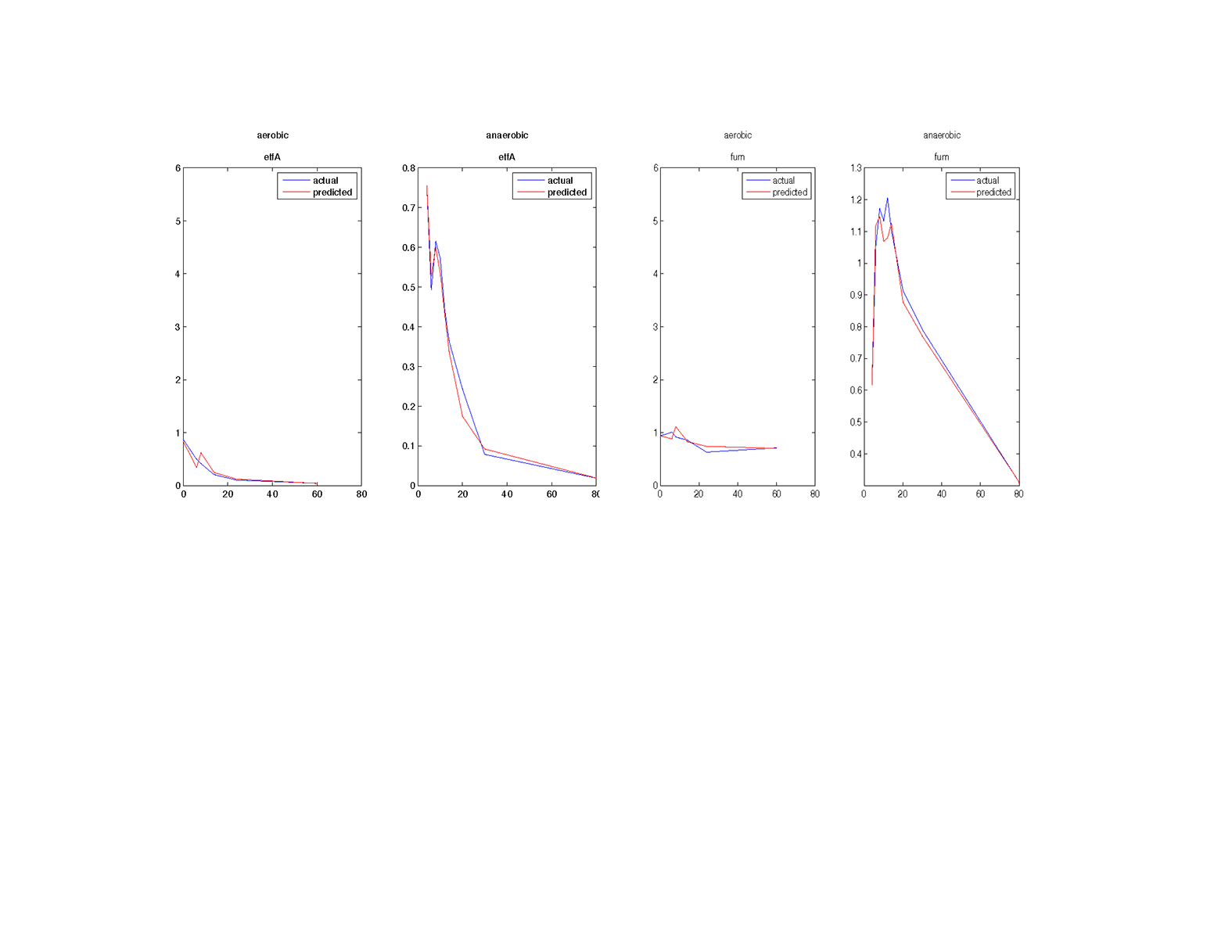

Supplement: Supplementary Figure 1 — Regression model for two genes etfA and fum: actual (blue) vs. predicted (red). [file Image1.TIFF]

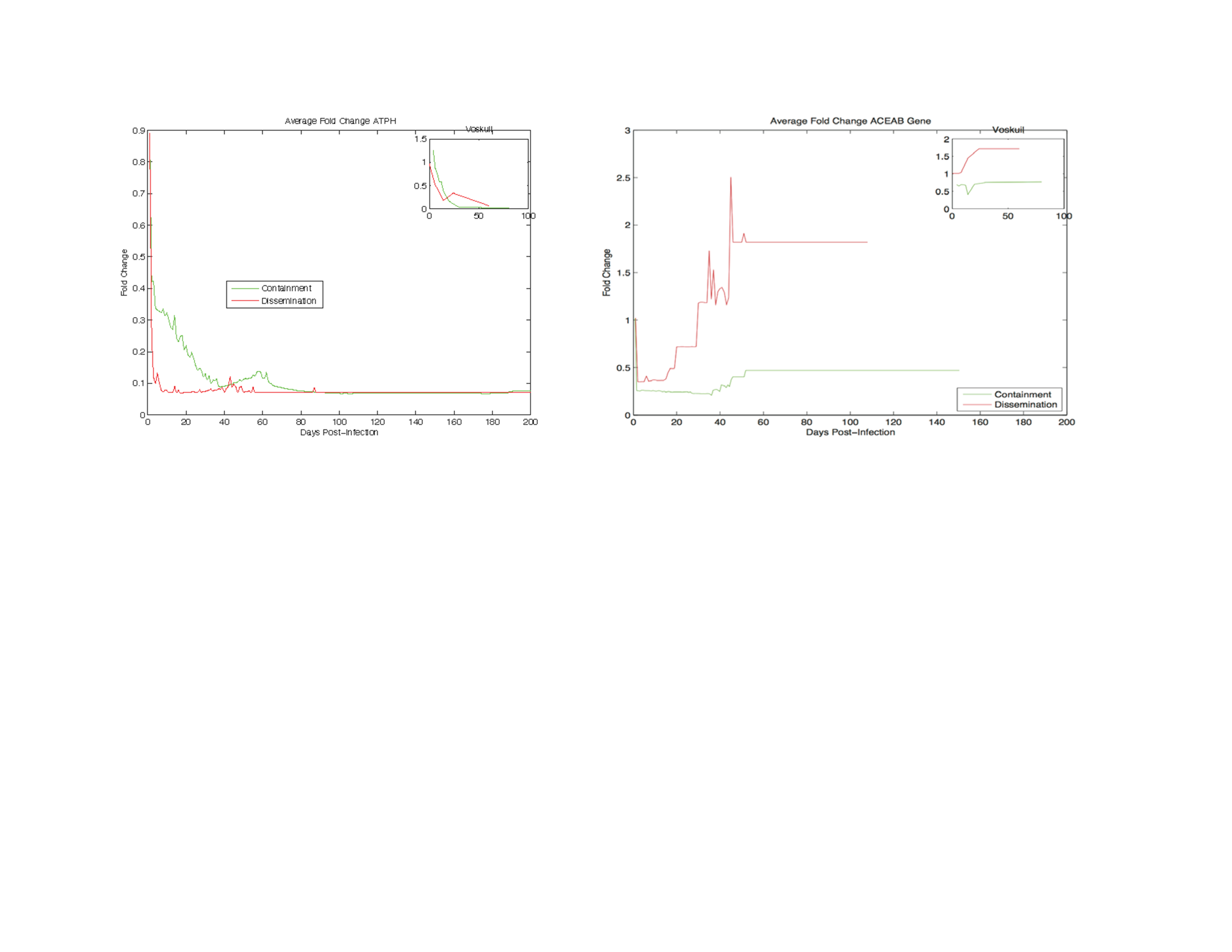

Supplement: Supplementary Figure 2 — Gene expression for cell at the center of the grid for the containment and dissemination outcome scenarios over 200 days post-infection. ATPH gene (Left); ACEAB gene (Right). Inset: Voskuil data for aerobic (red) and hypoxic (green) conditions. Results represent averages over 10 simulations per outcome (5 from N = 300 simulation run set and 5 from fixed parameter simulation runs). [file Image2.TIFF]

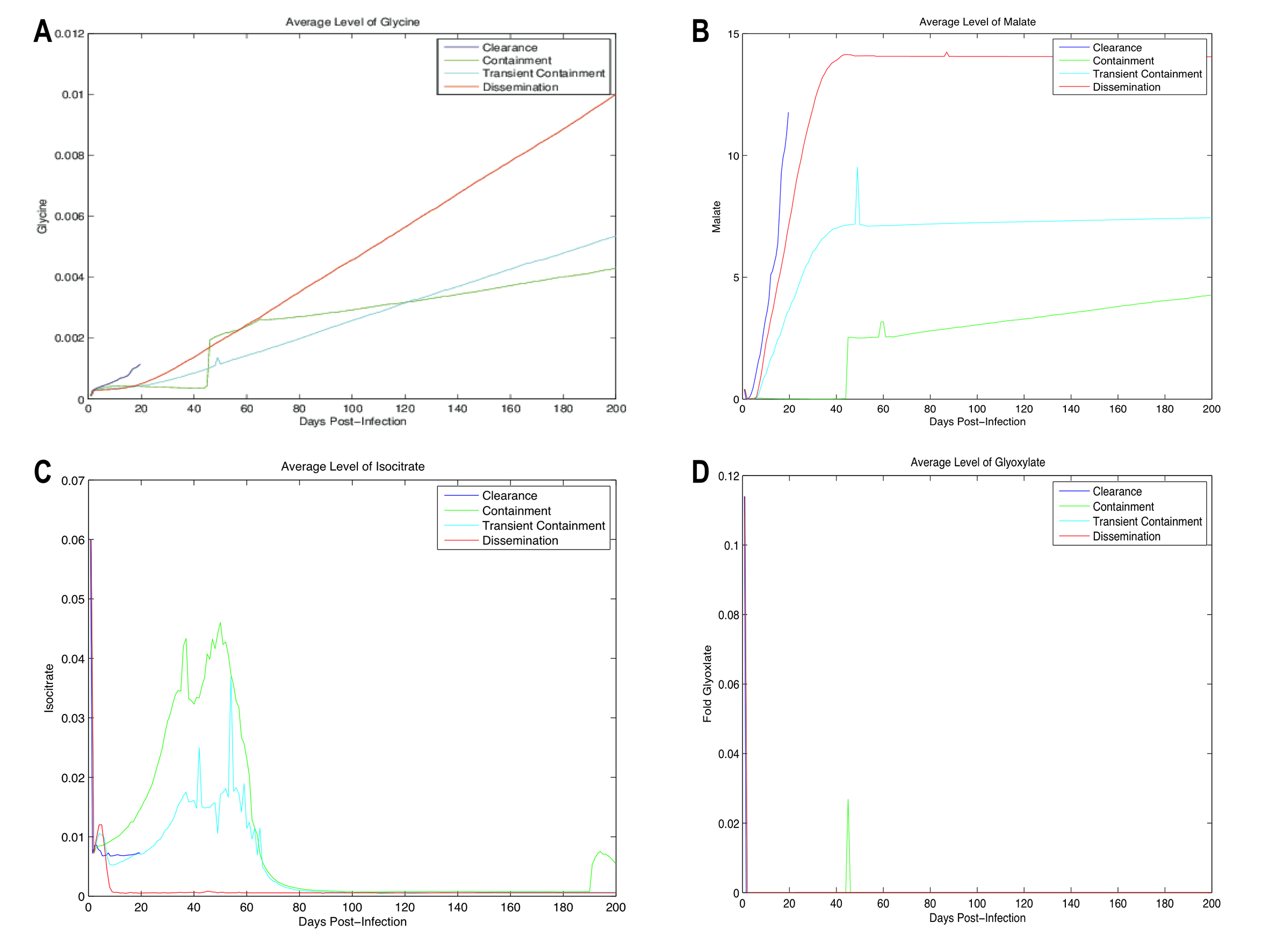

Supplement: Supplementary Figure 3 — Substrates levels from the Mtb metabolic model 200 days post-infection. Glycine-a; Malate-b; Isocitrate-c; Glyoxylate-d. Results represent sample averages from 300 simulation run set (number of simulations per outcome: 13 clearance, 6 containment, 5 transient containment, and 7 dissemination). [file Image3.tiff]

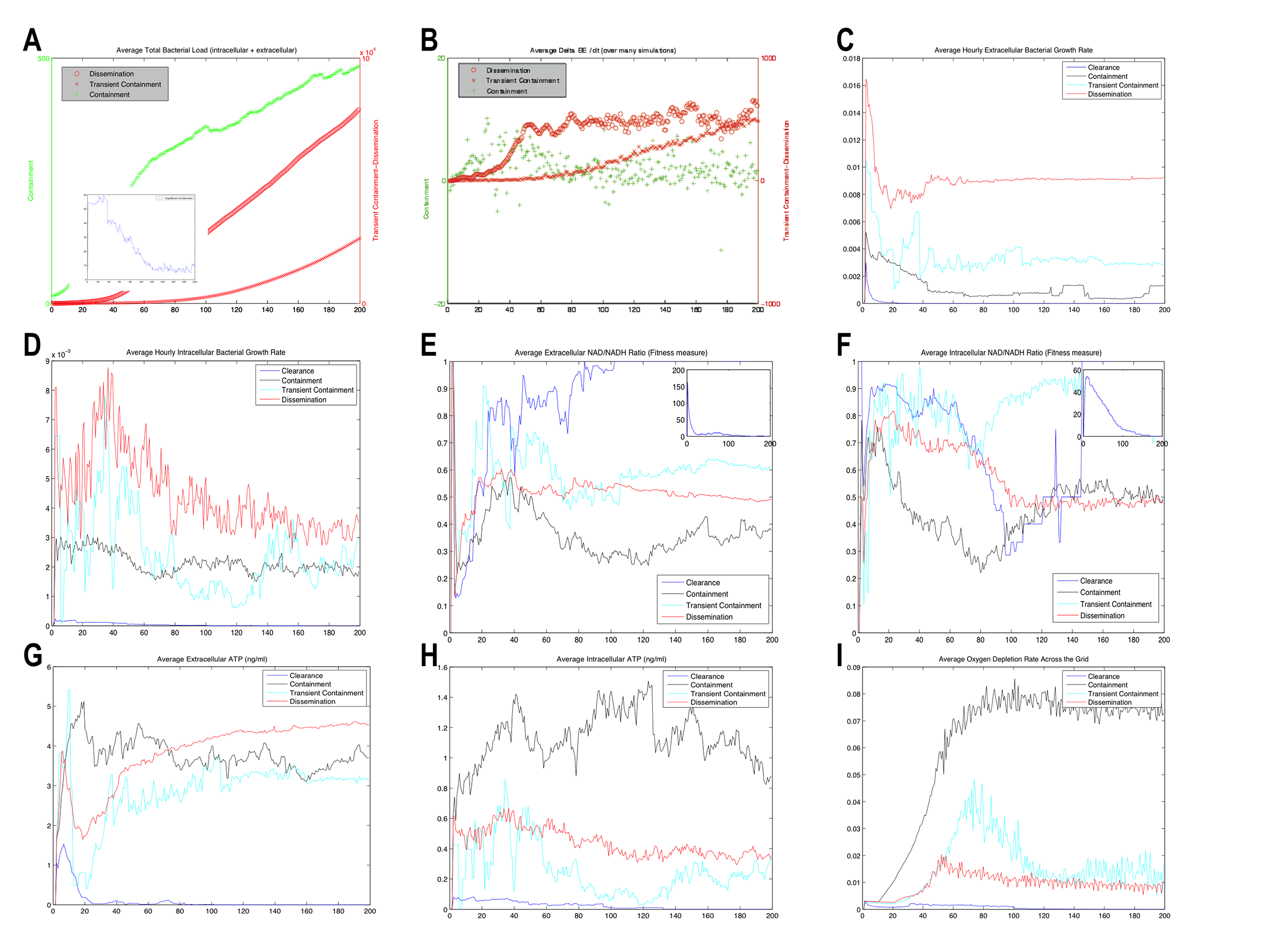

Supplement: Supplementary Figure 4 — Average response of cells across the simulation grid during clearance, containment, and dissemination. (A) Average growth rate of extracellular bacteria (inset - equilibrium containment averages); (B) average growth rate of intracellular bacteria; (C) average hourly extracellular growth rate; (D) average hourly intracellular bacterial growth rate; (E) average scaled NAD/NADH ratio of extracellular bacteria; (F) average scaled NAD/NADH Ratio of intracellular bacteria; (G) average extracellular ATP; (H) average intracellular ATP; (I) average rate of oxygen depletion across the granuloma. Averages over 300 total simulations. [file Image4.tiff]

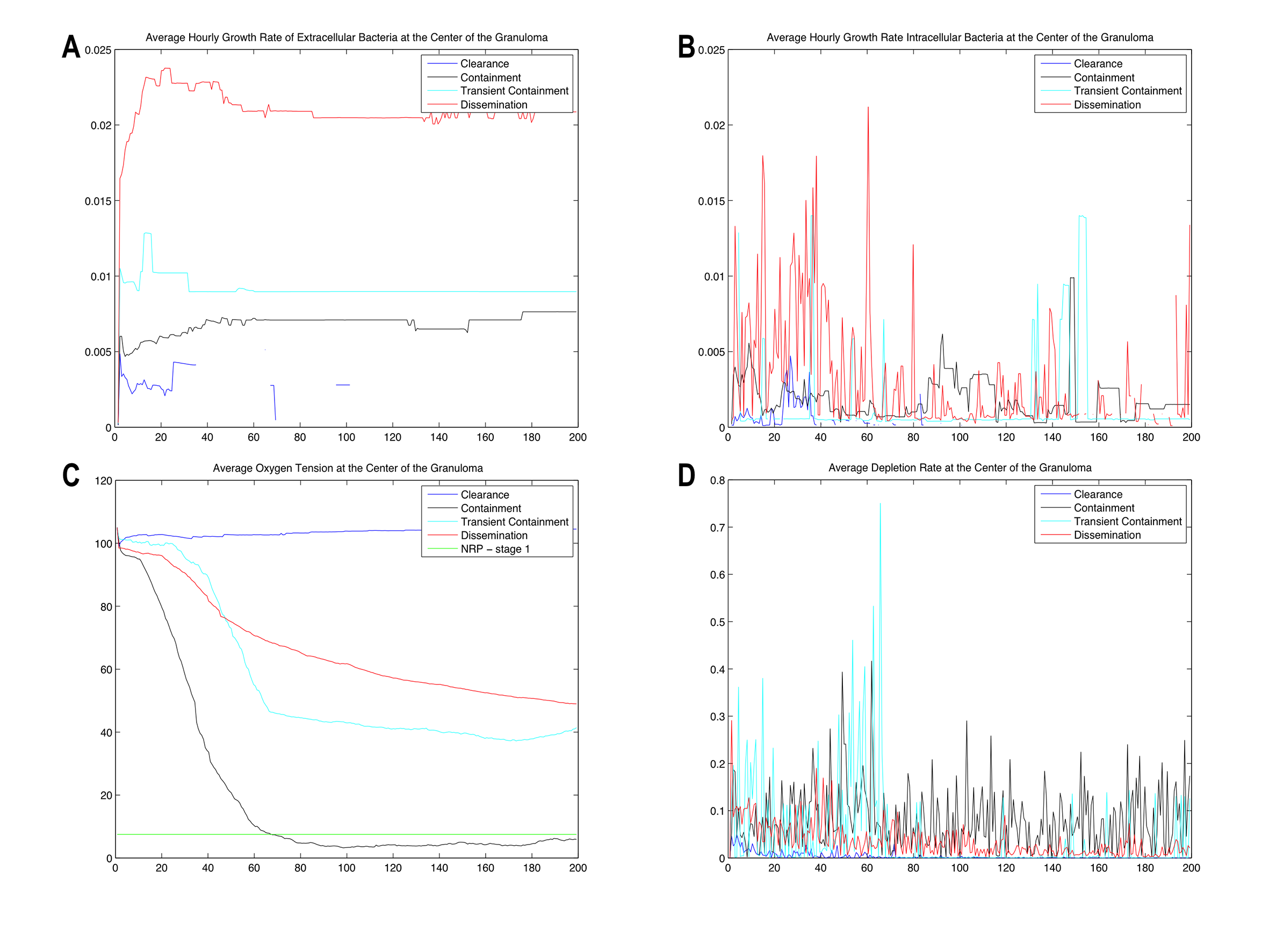

Supplement: Supplementary Figure 5 — Response of cell at the center of the grid for the clearance, containment, and dissemination outcome scenarios: (A) average growth rate of extracellular bacteria; (B) average growth rate of intracellular Bacteria; (C) average oxygen tension (mmHg); (D) average depletion rate. Averages over 300 total simulations. [file Image5.tiff]

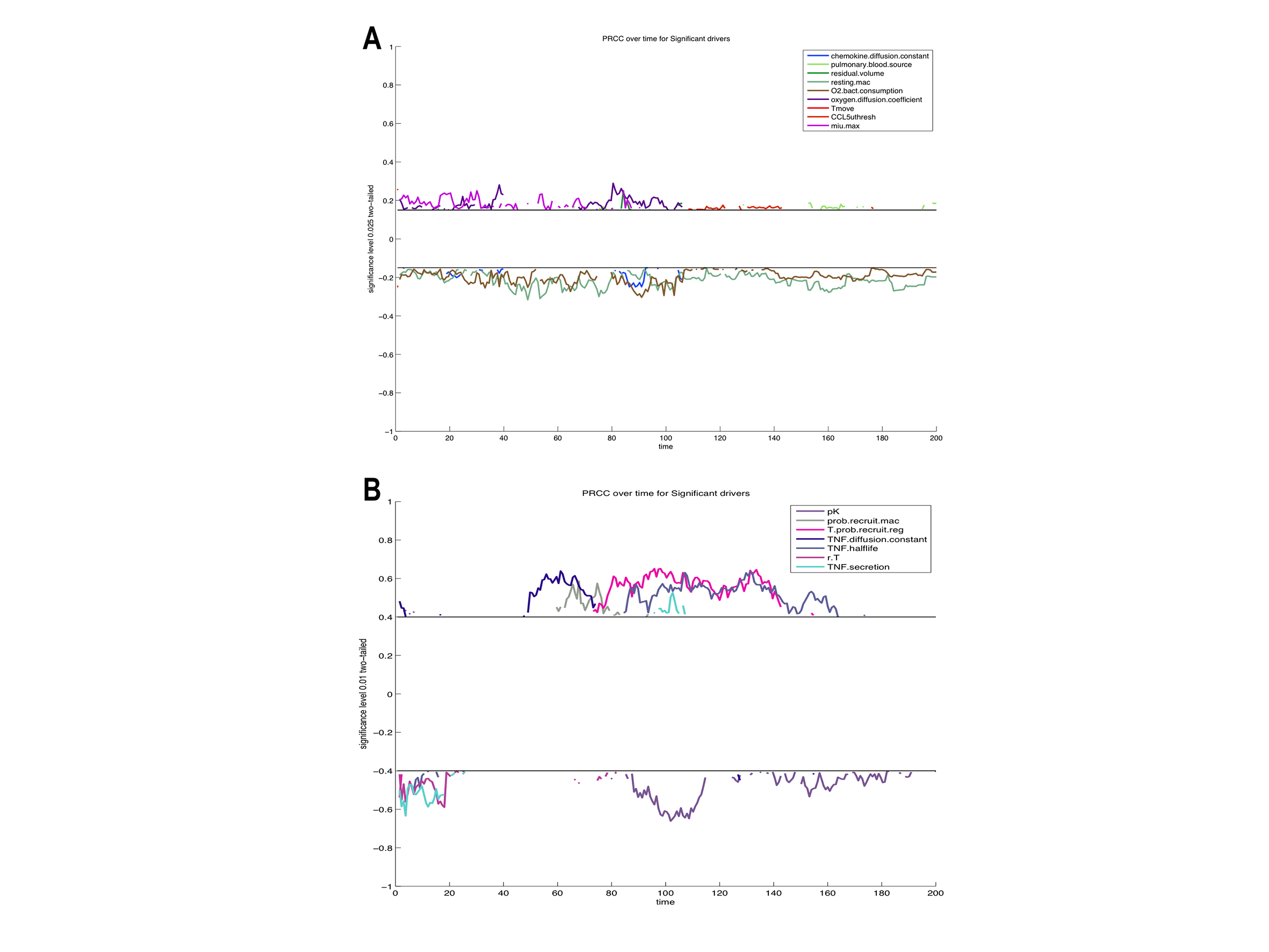

Supplement: Supplementary Figure 6 — Significant Partial Rank Correlation Coefficients for the integrated multiscale model show the impact of oxygen dynamics and physiological host response on Mtb infection outcome. Extracellular Bacterial levels are used as the outcome measure. Analysis results are based on multi-sample averages of the 300 simulation outcomes (A) and 41 individual non-clearance outcomes (B). [file Image6.tiff]

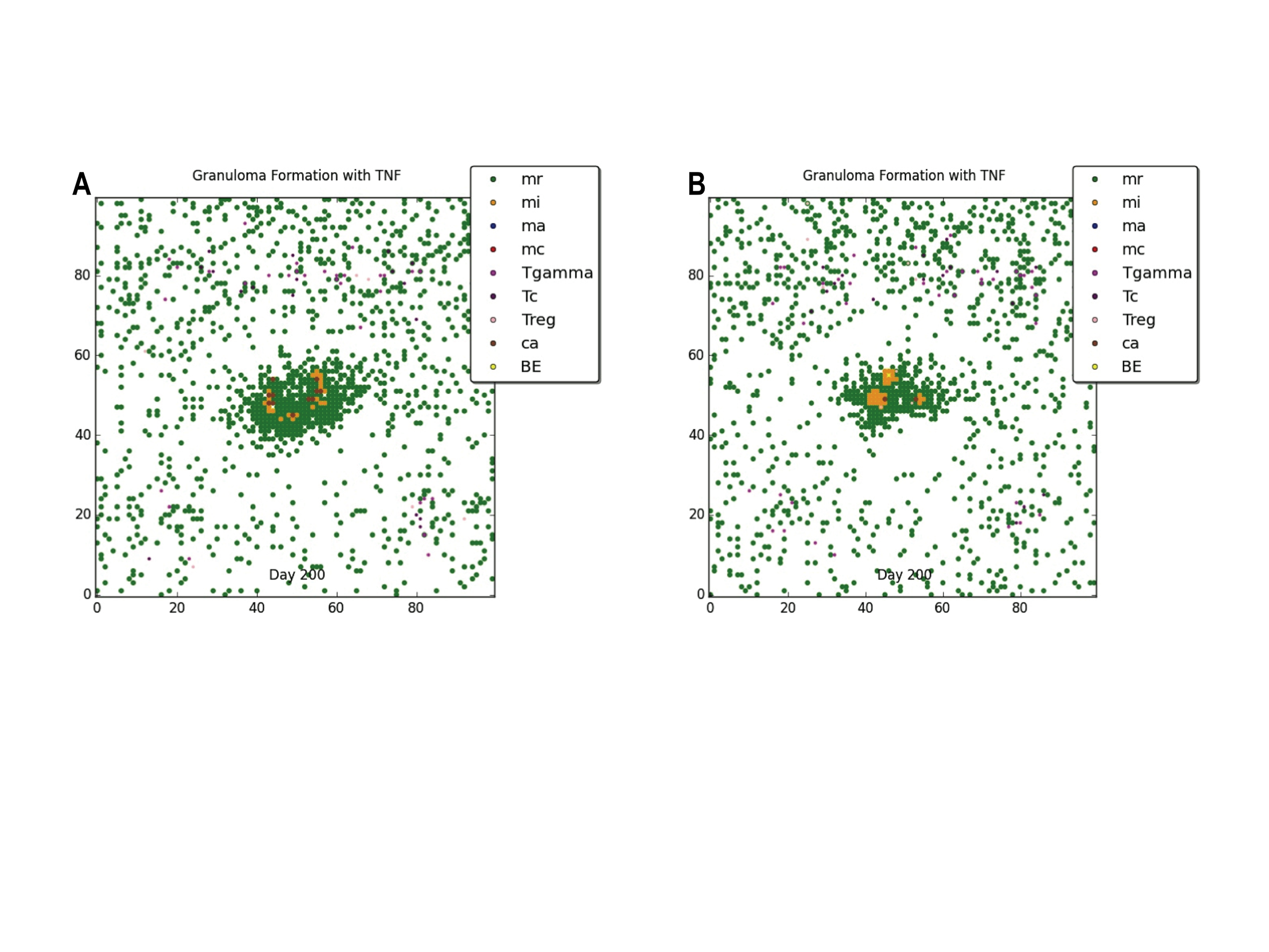

Supplement: Supplementary Figure 7 — Containment granuloma at 21 percent oxygen (left, A) and 18 percent oxygen (right, B). [file Image7.tiff]
