# Supplementary material for: Oxygen Modulates the Effectiveness of Granuloma Mediated Host Response to Mycobacterium tuberculosis: A Multiscale Computational Biology Approach
Source: Front Cell Infect Microbiol. 2016 Feb 15;6:6. doi: 10.3389/fcimb.2016.00006 (PMC4753379; doi:10.3389/fcimb.2016.00006)
Supplement: Supplementary Table 1 — Parameters of the containment and dissemination run both with the ABM as standalone model and the multi scale model. [file DataSheet4.pdf]

Table 1. Appendix 4: Parameters of the containment and dissemination run both with the ABM as standalone model and the multi scale model

Variable	Containment (fig 3e and 3f)	Dissemination (fig 4)
Mtb doubling time (hours)	116.67	88
initial number of macrophages	120	99
probability a resting macrophage kills a bacteria	0.1	0.05
macrophage TNF detection threshold (molecules)	2.50E+05	n/a
probability a resting macrophage moves into the same compartment as a T cell	0.95	0.0526
number of burstings for a cell to become caseous	8	8
lower threshold for chemokine biased move molecules	1.41E+04	1.41E+04
upper threshold for chemokine biased move molecules	1.55E+04	1.55E+04
probability a mac will be recruited at a vascular source	0.075	0.04
time delay for recruitment (days)	10	20
No. of T cells needed to activate a macrophage	4	6
max lifetime (days) of activated macrophage	10	10
Intracellular bacteria growth rate per minute	0.00015	0.00044
No. of intracellular bacteria defining transition to chronically infected state	10	10
probability a T cell will activate a macrophage	0.1	0.05
level of TNF necessary to activate macrophage	2.50E+05	n/a
probability of TNF-independent apoptosis by T cells	0.006	0.0631
O2 residual volume in tissue from pulmonary blood volume ml/m^2/3.5sec	634	504
O2 residual volume in tissue in liters	2.75	1.20
hourly maximum specific growth rate of bacteria	0.006649	0.008
Intracellular max growth rate per hour (2 * extracellular growth rate per hour)	1.5*μ_{max}	2*μ_{max}
TNFα molecules secreted hourly	3.15E+06	0
CCL5 molecules secreted hourly	4.50E+06	4.50E+06
threshold for playing a role with IFN-gamma activation of macrophages	50	100

Parameters not listed here are the defaults in table 3.
