# Supplementary material for: Oxygen Modulates the Effectiveness of Granuloma Mediated Host Response to Mycobacterium tuberculosis: A Multiscale Computational Biology Approach
Source: Front Cell Infect Microbiol. 2016 Feb 15;6:6. doi: 10.3389/fcimb.2016.00006 (PMC4753379; doi:10.3389/fcimb.2016.00006)
Supplement: Supplementary file 9 [file DataSheet1.PDF]

Appendix 1.1: Algorithm for categorizing infection outcomes

- **Clearance: If at any timestep, total bacteria (extracellular, intracellular, and bacteria residing in caseous regions) is 0, then classify the outcome as infection clearance.**

- **Containment:** If not cleared, then use derivatives Delta_BE/dt and Delta_mr/dt where BE is extracellular bacteria and mr is the number of resting macrophages recruited to determine if the model results in containment. If -5 < mean (Delta_BE/dt) over 200 days < 5 and macrophage recruitment is increasing over the interval (22 days, 50 days) (Delta mr / dt positive) then classify as containment.

- **Transient Containment:** If not clearance and not containment, then use derivatives to characterize if the model results in transient containment. If -25< mean(Delta_BE/dt) over days 1-70 <25 and macrophage recruitment is increasing over the interval (22 days, 50 days) (Delta mr / dt positive) (see Figure 5) then classify as transient containment.

- **Dissemination:** If neither of the conditions above is met, then what remains are the qualitative outcomes that are classified as dissemination.

Appendix 1.2: Rules governing macrophage behavior under hypoxic conditions

- Track the time the macrophage has spent under hypoxic or anoxic conditions.
- If time spent in hypoxia/anoxia is < limit (48 hours, hypoxia; 12 hours anoxia), there is some probability (0.001-0.01) of apoptosis per 18-hour interval.
- If macrophage survives after limit is exceeded, macrophage is immune to hypoxia/anoxia (up-regulation of oxygen regulated protein under hypoxia may convey protection from apoptosis), but macrophage may die from natural causes.
