# Supplementary material for: Oxygen Modulates the Effectiveness of Granuloma Mediated Host Response to Mycobacterium tuberculosis: A Multiscale Computational Biology Approach
Source: Front Cell Infect Microbiol. 2016 Feb 15;6:6. doi: 10.3389/fcimb.2016.00006 (PMC4753379; doi:10.3389/fcimb.2016.00006)
Supplement: Supplementary file 10 [file DataSheet2.PDF]

Appendix 2: Rules for solving the steady state solution for the oxygen field:

We wish to solve *D**Av=b where v is the solution vector of the number of molecules of oxygen available in tissue for a particular grid cell. Here the vector b is the vector of source/sinks (in terms of the number of molecules) and is of size x*y, where x is the number of cells in the grid in the horizontal direction and y is the number of cells in the grid in the vertical direction. The matrix A is the two-dimensional, square symmetric positive definite 5-point laplacian of size (x*y) X (x*y).

1) Initialize the vector b to zero.

2) Place the sources and sinks into the b vector. Sources are sources of oxygen like vascular source sites and boundary cells and sinks are consumers of bacteria like macrophages and bacteria and T cells.

3) Determine how many oxygen molecules are in tissue at the source (boundary) cells, assuming that there is no depletion anywhere else in the lung. For example we can use the ideal gas law (PV=nrt) and calculate residual volume:

- The average lung contains 1.2 liters of air, and normal air contains 21% oxygen, so 252 mL of O2 are in the residual volume of the human lung. Of this amount 25% is transferred to tissue [41], so a volume of 63 mL O2 is transferred to tissue per 1200mL of air. Now apply the ideal gas law to find the number of molecules permeating the tissue and spread over the entire surface area of the human lung (~130 m^2 [47])to calculate the number of molecules available in each grid site.
- Oxygen molecules sourced from the pulmonary blood volume use PV = nrt, to convert to number of molecules per grid cell as explained above.

4) Calculate the O2 consumption by the consumers (sinks). As an example:

- A resting macrophage consumes 1.15 micromoles/ 10^7 cells/ hr [23]. We directly calculate the number of molecules consumed by a macrophage per 4 seconds (1 breath).
- Other consumers are activated macrophages, infected macrophages, chronically infected macrophages, T cells, and bacteria.

5) Find a suitable diffusion coefficient for oxygen in lung tissue (see Table 1 for range used).

6) Scale the terms of the Laplacian and the boundary terms of *b* by *D*/h^2 (order of the second degree error term).

7) Use NumPy, Matlab or Octave to solve for the steady-state vector x

8) Reshape the resulting solution vector v into the lexicographically-ordered-by-y-lines x X y grid.

9) Plot the steady-state diffusion portrait for this point in time.
