# Supplementary material for: Oxygen Modulates the Effectiveness of Granuloma Mediated Host Response to Mycobacterium tuberculosis: A Multiscale Computational Biology Approach
Source: Front Cell Infect Microbiol. 2016 Feb 15;6:6. doi: 10.3389/fcimb.2016.00006 (PMC4753379; doi:10.3389/fcimb.2016.00006)
Supplement: Supplementary file 11 [file DataSheet3.PDF]

Appendix 3: Incorporating the intracellular and extracellular models via BioXyce: gene expression

Initialize gene/substrate files for all new grid cells and/or newly infected macrophages

Calculate gene expression for 59 genes that figure into the intracellular model

a. Dependent variable: gene expression
b. Independent variables
    i. Change in time, change in time squared
    ii. Change in percent oxygen in tissue
    iii. Depletion rate (change in O2/change in time)
    iv. ATP, ATP squared
c. Range R squared: 0.53 – 0.942; average: 0.80

Write input parameter file for gene expression values and current oxygen levels

For every grid cell containing bacteria, run intracellular model with BioXyce and output new substrate values

For every macrophage containing bacteria run intracellular model with BioXyce and output the new substrate values

ATP is used by the update routine to determine the current growth rate based upon O2 levels via the Monod Equation with death rate modulated by the normalized NAD/NADH ratio
